# PEGylated Gold Nanoparticles Target Age-Associated B Cells In Vivo

**DOI:** 10.1021/acsnano.2c04871

**Published:** 2022-10-27

**Authors:** Sandra Hočevar, Viola Puddinu, Laetitia Haeni, Alke Petri-Fink, Julia Wagner, Montserrat Alvarez, Martin James David Clift, Carole Bourquin

**Affiliations:** † Institute of Pharmaceutical Sciences of Western Switzerland, University of Geneva, Geneva 1211, Switzerland; ‡ Department of Anaesthesiology, Pharmacology, Intensive Care and Emergency Medicine, Faculty of Medicine, University of Geneva, Geneva 1211, Switzerland; § BioNanomaterials, Adolphe Merkle Institute, University of Fribourg, Fribourg 1700, Switzerland; ∥ In Vitro Toxicology Group, Swansea University Medical School, Swansea, Wales SA2 8PP, U.K.

**Keywords:** gold nanoparticles, polymer-coating, in vivo toxicity, targeted delivery, B cells, age-associated B cells

## Abstract

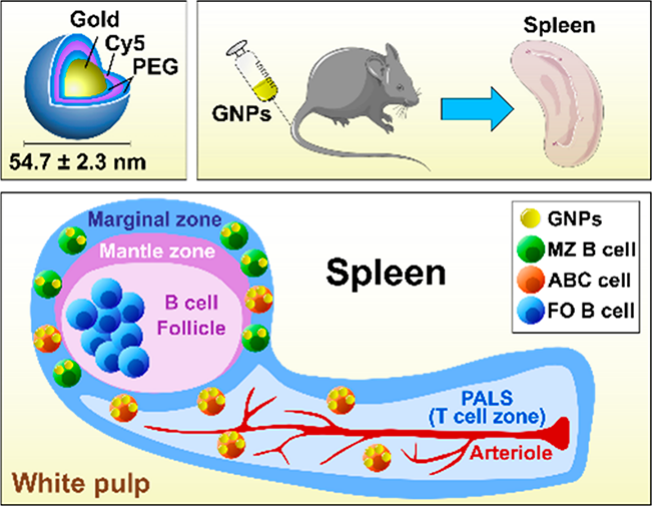

Engineered gold nanoparticles (GNPs) have become a useful tool in various therapeutic and diagnostic applications. Uncertainty remains regarding the possible impact of GNPs on the immune system. In this regard, we investigated the interactions of polymer-coated GNPs with B cells and their functions in mice. Surprisingly, we observed that polymer-coated GNPs mainly interact with the recently identified subpopulation of B lymphocytes named age-associated B cells (ABCs). Importantly, we also showed that GNPs did not affect cell viability or the percentages of other B cell populations in different organs. Furthermore, GNPs did not activate B cell innate-like immune responses in any of the tested conditions, nor did they impair adaptive B cell responses in immunized mice. Together, these data provide an important contribution to the otherwise limited knowledge about GNP interference with B cell immune function, and demonstrate that GNPs represent a safe tool to target ABCs *in vivo* for potential clinical applications.

## Introduction

Gold nanoparticles (GNPs) have gained increasing attention over the past two decades in the field of nanomedicine due to their intriguing properties. Among other fascinating applications, GNPs are used as carriers for drugs and therapeutics, and can serve as a convenient platform for vaccine delivery.^1^ Administration of GNPs conjugated with antigens of several pathogens has shown a significant improvement in the adaptive immune response, compared to immunization with soluble antigens.^2−5^ This is due to the B cell’s preference to phagocytize particulate antigens, which significantly enhances antigen presentation to T cells and leads to efficient activation of the cellular and humoral adaptive immune responses.^6,7^

Nevertheless, as the number of various GNP biomedical applications increases, questions about their safe use in humans have been raised; therefore, numerous studies have been conducted to evaluate the potential toxicity of GNPs and their adverse impact on the immune system. It has been reported that GNPs were able to increase serum pro-inflammatory cytokines which expanded T and B cells and impacted antigen presentation in dendritic cells.^8−10^ However, the presence and severity of these effects were strongly dependent on GNP physicochemical properties including size, shape, surface charge, polymer coating, colloidal (in)stability, and the lack of relevant/realistic exposure concentrations.^11,12^ Polymer coating of GNPs is a common practice to raise the level of biocompatibility and stability.^13^ In particular, coating with polyethylene glycol (PEG) improves GNPs half-life in blood as it prevents the formation of an outer protein corona.^14^ However, it was reported that PEGylated nanomedicines are not entirely resistant to opsonization with plasma proteins, such as immunoglobulins and complement proteins, which can potentially lead to receptor-mediated immune responses.^15,16^

Whereas the vast majority of studies regarding GNP immunotoxicity focus on myeloid cells, which typically are the main sub-populations to interact with GNPs due to their potent phagocytic properties, there is insufficient knowledge about the GNP impact on other cell types such as B cells. These immune cells play an important role in innate and adaptive immunity by generating antigen-specific immune responses. Due to their location and abundance in secondary lymphoid organs, such as the spleen and the lymph nodes (LN), B cells are a likely target following GNP administration. In fact, systemically administered GNPs with a diameter of ∼50 nm were detected in the majority of the organs but had a stronger tendency to accumulate in the liver and spleen over time.^17^ Moreover, the subcutaneous injection of NPs smaller than 100 nm showed a passive draining and accumulation in the LN.^18^ For these reasons, this study aims to elucidate the potential interactions of PEGylated GNPs with B lymphocytes *in vivo*.

Historically, three B cell sub-populations have been described, differing in origin, localization, phenotype, and functions. These populations were divided into follicular (FO) B cells, marginal zone (MZ) B cells, and B-1 B cells. However, during the past decade, age-associated B cells (ABCs) have been identified as a forth subset of B cells with distinct phenotypical and functional characteristics.^19,20^ ABCs play a role in several contexts including aging, response to microbes, and autoimmunity.^21^ Similarly to MZ B cells, ABCs reside mainly in the spleen at the borders of the B cell and T cell zone,^22^ where they can encounter blood-borne antigens and are able to secrete antibodies in a TLR7/9- or IL21-mediated manner.^23,24^ ABCs differ from FO B cells and MZ B cells on different accounts including origin, mode of activation, and effector functions, as elegantly reviewed by Mouat,^25^ Cancro,^21^ and Ma et al.^26^

Here we investigate how polymer-coated GNPs interact with B cells in different organs and different immune cell subsets in mice. Importantly, the data indicate how GNPs affect B cell functions *in vivo* in the presence of either an antigen or an adjuvant. Thus, these data provide important evidence toward the potential applications of GNPs for biomedical applications, in particular for targeting specific sub-populations of B cells.

## Results and Discussion

### Polymer-Coated GNPs Are Stable in Different Biological Media

To investigate the interactions of GNPs with B cells and to follow their biodistribution in mice, fluorescently labeled gold nanospheres coated with polyethylene glycol (PEG) were synthesized as previously reported by Rodriguez-Lorenzo et al. and characterized by Hočevar et al.^27,28^ This GNP formulation consisted of two layers of PEG polymer. The first layer of PEG coating (NH_2_-PEG) was conjugated with cyanine 5 (Cy5) fluorochrome, followed by a second layer of PEG in order to shield Cy5 and to prevent a potential interaction of Cy5 chemistry with the biological system (Figure S1A).^27^ The average hydrodynamic diameter of GNPs was 54.7 ± 2.3 nm, measured by dynamic light scattering (DLS), whereas the zeta potential was 13.4 ± 1.5 mV, both measured in water (pH ∼ 7). The double PEGylated Cy5-labeled GNPs were used throughout this study.

The colloidal stability of NPs can significantly change after contact with biological media and result in, amongst others, NP aggregation.^29^ This can lead to changes in NP internalization by cells and consequently increase the risk of NP toxicity.^30^ To evaluate the GNP colloidal stability, GNPs were incubated in different biological media (H_2_O, PBS, cell culture medium with 10% FBS, 15% mouse serum) overnight at 37 °C. UV–vis measurements showed that polymer-protected GNPs did not aggregate in biological media, as all samples absorbed light at ∼520 nm (Figure S1B), which is a typical absorption peak for stable 15 nm gold nanospheres.^31^ Minor changes were observed only on mouse serum. Thus, we confirmed high colloidal stability of polymer-protected GNPs even in protein-rich environments such as cell culture medium.

### GNPs Accumulate in B Cells of the Spleen after Short-Term Exposure

**Figure 1:**
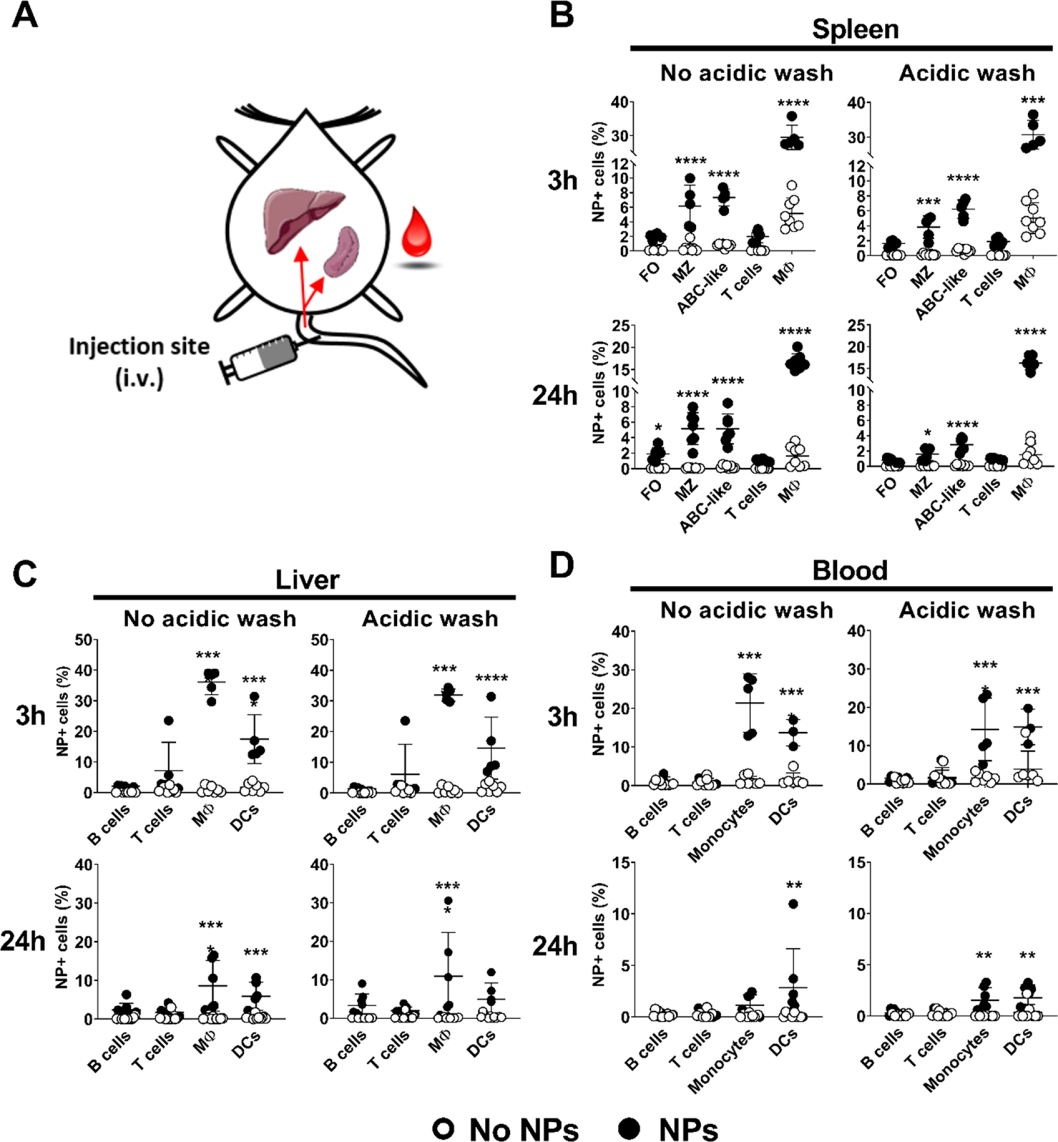
Biodistribution of GNPs after intravenous injection. (A) Scheme of the GNP administration and targeted organs (spleen, liver, and blood) in C57/BL6 mice. Total GNP-cell association (no acidic wash) and uptake of GNPs (acidic wash) were measured in different immune cell populations in the (B) spleen, (C) liver, and (D) blood 3 and 24 h after intravenous injection of 400 μg GNPs in the tail vein. The acidic wash was performed prior to cell staining and measurement of GNP-Cy5 positive cells by flow cytometry. Data for each time point are pooled from two separate experiments. Each dot represents one mouse (*n* = 5–8). Error bars: mean ± SD of the pooled data; **p < 0.01, ***p < 0.001, ****p < 0.0001. Data were evaluated by two-way ANOVA, followed by Sidak’s multiple comparison post hoc test. B cells (CD19^+^/CD3^–^), MZ: marginal zone B cells (CD19^+^/CD21^+^/CD23^–^), FO: follicular B cells (CD19^+^/ CD3^–^/CD21^+^/CD23^+^), ABC-like B cells (CD3^–^/CD19^+^/CD21^–^/CD23^–^), T cells (CD19-/CD3+), macrophages (Mφ)/monocytes (CD11b^+^/CD11c^–^), dendritic cells (DC): (CD11b^±^/CD11c^+^). Gating strategy, see Figure S8.

To determine GNP biodistribution in mice and to examine whether the GNPs physically interact with B cells in different organs, 400 μg of GNPs was injected intravenously. This quantity is in the high dose range and ensured sufficient accumulation of GNP in the organs, favoring potential interactions with B cells.^17,32^ After 3 or 24 h, spleen, liver, and blood were collected and processed to determine cellular uptake of GNPs with flow cytometry by means of Cy5 fluorescence (Figure 1A). Surface association and internalization of GNPs were differentiated after incubation of cells with an acidic wash (∼pH 4), which causes a detachment of membrane-bound molecules.^33^ This method, previously employed by us and others,^28,34^ causes cell death of 10% of cells, without preferential killing of any sub-population (data not shown). As expected, GNPs were highly internalized by phagocytic cells (*e.g.*, monocytes, macrophages, DCs) in all the tested organs, and GNP^+^ cells quickly declined in blood after 24 h (Figure 1). On the contrary, T cells did not associate with GNPs in any of the organs examined (Figure 1). We observed that GNPs were internalized by specific sub-types of splenic B cells after 3 and 24 h. These populations were MZ B cells and ABC-like B cells. The latter were identified based on the phenotype reported by Hao et al. and Rubtsov et al., where ABCs are either CD19^+^/CD21^–^/CD23^–^ or CD19^+^/CD11c^+^.^19,20^ In contrast to ABC-like and MZ B cells, follicular cells only bound GNPs to the cell surface, as their association was lost after the acidic wash (Figure 1B). Due to the architecture of the spleen, MZ B cells and ABC-like B cells localized in the marginal zone,^22,35^ which surrounds B cell follicles and is exposed to the blood circulation. Consequently, both MZ B cells and ABCs are most likely the first splenic B cell sub-types to interact with the GNPs after intravenous injection. In contrast, GNPs did not associate with B cells present in the liver and blood (Figure 1C and 1D).

**Figure 2:**
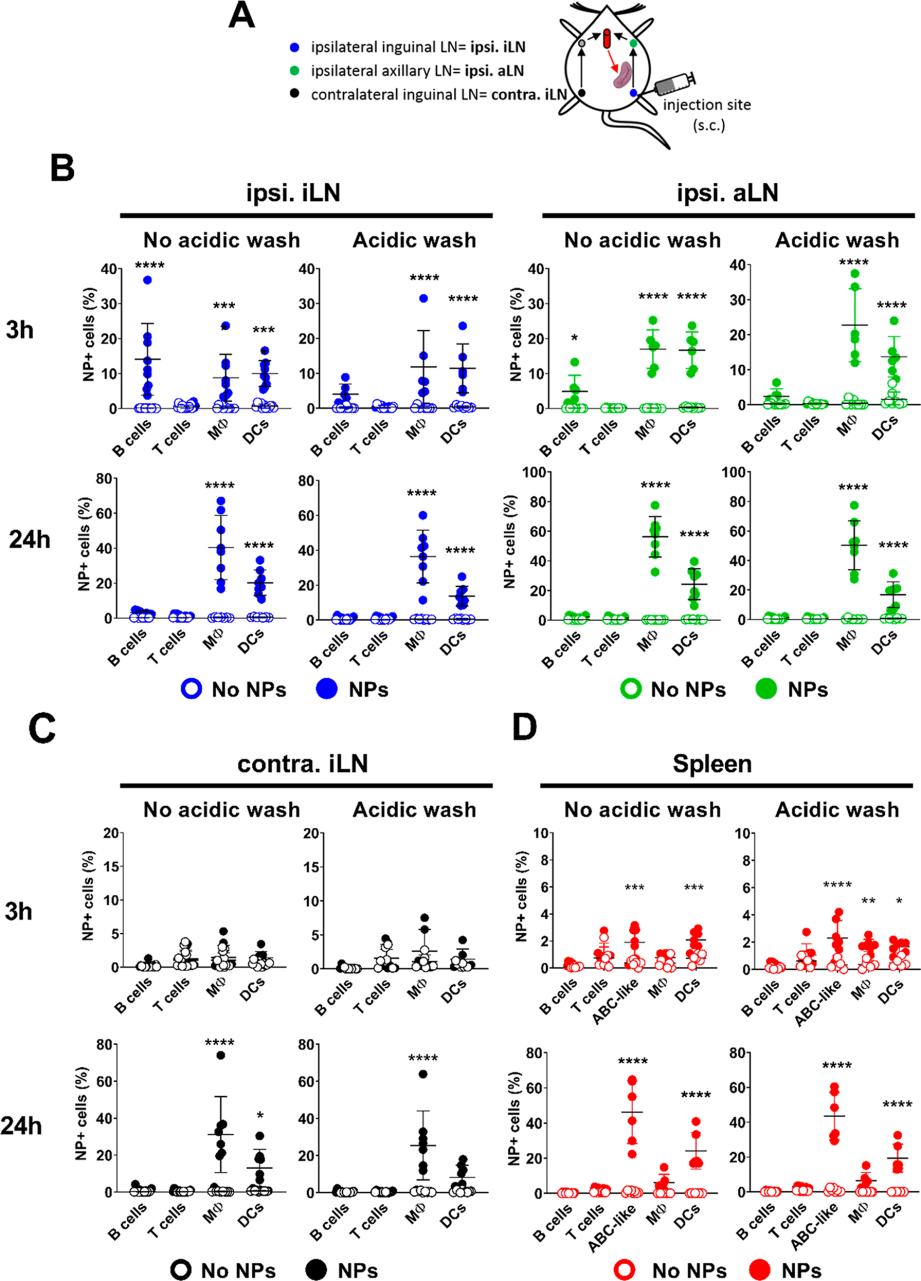
Biodistribution of GNPs after subcutaneous injection. (A) Scheme depicting GNP administration and targeted LN, based on their position in relation to the site of injection in C57/BL6 mice. Mice were injected sub-cutaneously in the flank with 300 μg GNPs and exposed for 3 or 24 h. Total GNP-cell association (no acidic wash) and uptake of GNPs (acidic wash) were measured in different immune cell populations in the (B) ipsilateral inguinal LN (ipsi. iLN), ipsilateral axillary LN (ipsi. aLN), (C) contralateral inguinal LN (contra. iLN), and (D) spleen. Data for each time point are pooled from two to three separate experiments. Each dot represents one mouse (*n* = 6–12). Error bars: mean ± SD of the pooled data. Data were evaluated by two-way ANOVA, followed by Sidak’s multiple comparison post hoc test. *p < 0.05, ***p < 0.001, ****p < 0.0001. B cells (CD19^+^/CD3^–^), T cells (CD19^–^/CD3^+^), ABC-like B cells (CD3^–^/CD11c^+^/CD11b^–^/CD19^+^), Mφ/monocytes (CD11b^+^/CD11c^–^), DC: (CD11b^±^/CD11c^+^/CD19^–^).

In order to target B cells in the LN, GNPs were subcutaneously injected (300 μg) into the flank of the mouse. After 3 or 24 h, the primary draining LN (ipsilateral inguinal LN, ipsi. iLN), the secondary draining LN (ipsilateral axillary LN, ipsi. aLN), or the nondraining LN (contralateral inguinal LN, contra. iLN) and spleen were analyzed (Figure 2A). The results suggest that GNPs were transiently internalized by B cells in one of the draining LNs 3 h after injection. Furthermore, GNPs shortly associated (no acidic wash) with the B cells only in the draining LNs (Figure 2B and 2C). Again, macrophages, followed by DCs, were the leading cells to internalize GNPs in the draining, non-draining LNs and in the spleen (Figure 2B, 2C, and 2D). The lack of uptake from the B cells in the lymph nodes and in the other tested organs can be explained by the fact they lack the major GNP-interacting B cell sub-sets, as MZ and ABC-like cells specifically reside in the spleen. In this last mentioned organ, GNPs were detected in phagocytes (DCs and macrophages) as early as 3 h after sub-cutaneous injection, indicating that GNPs quickly enter the systemic circulation (Figure 2D). Surprisingly, ABC-like B cells internalize GNPs already at this time point, with uptake further increased at 24 h, where GNPs can be detected only in DCs and ABC-like cells (Figure 2D). Taken together, the level of GNP biodistribution differed among immune cell populations and their sub-sets in different organs, and was dependent on the time of exposure and route of administration.

### Polymer-Coated GNPs Are Highly Immunocompatible

To examine the overall biocompatibility of intravenously injected GNPs, cell viability was measured by flow cytometry, using an amine-reactive viability dye. The results showed no decrease in cell viability among splenocytes, lymph node cells, blood, and liver leukocytes up to 24 h after intravenous (Figure S2A and SC) or sub-cutaneous injection (Figure S3A and S3B). Moreover, no significant changes in frequencies of immune cells and B cell populations (MZ, FO, and ABC-like B cells) were observed after 3 and 24 h (Figure S2B, S2D, S3A, and S3B). For the gating strategy, see Figure S9.

Some studies have reported the ability of GNPs to induce an immune response, resulting in the expression of activation markers and production of pro-inflammatory cytokines and antibodies.^36−38^ To evaluate whether the GNPs could activate B cells in mice, the expression of a common immune cell activation marker, CD86, and the production of IgM and IgG antibodies were examined upon intravenous or sub-cutaneous injection of GNPs. GNPs did not cause any increase in CD86 expression in B cells or other immune cells (macrophages/monocytes, DCs) in any of the organs tested up to 24 h (Figure S2E and S3C). Moreover, it was observed that GNPs did not cause an increase in serum IL-6 (Figure S2F and S3D) nor impacted the levels of total IgM and IgG in the serum up to 24 h postinjection (Figure S2G and S3E).

In order to investigate how LN and splenic immune cells are directly affected by GNPs, primary mouse splenocytes or LN cells were incubated with high concentrations of GNPs (20 μg/mL) for 24 h at 37 °C, 5% CO_2_. Despite these high GNP concentrations and high GNP-cell association *in vitro* (Figure S4A), no change in viability, frequency of immune sub-populations, or increase in IL-6 release was detected (Figure S4B, S4C, and S4D). Together, these *in vivo* and *in vitro* results suggest a high biocompatibility of GNPs, with no adverse effect on cell viability or induction of B cell activation in adjuvant- and antigen-free conditions.

### GNPs at High Concentrations Impair Function of Adjuvant-Activated B Cells *In Vitro*

**Figure 3:**
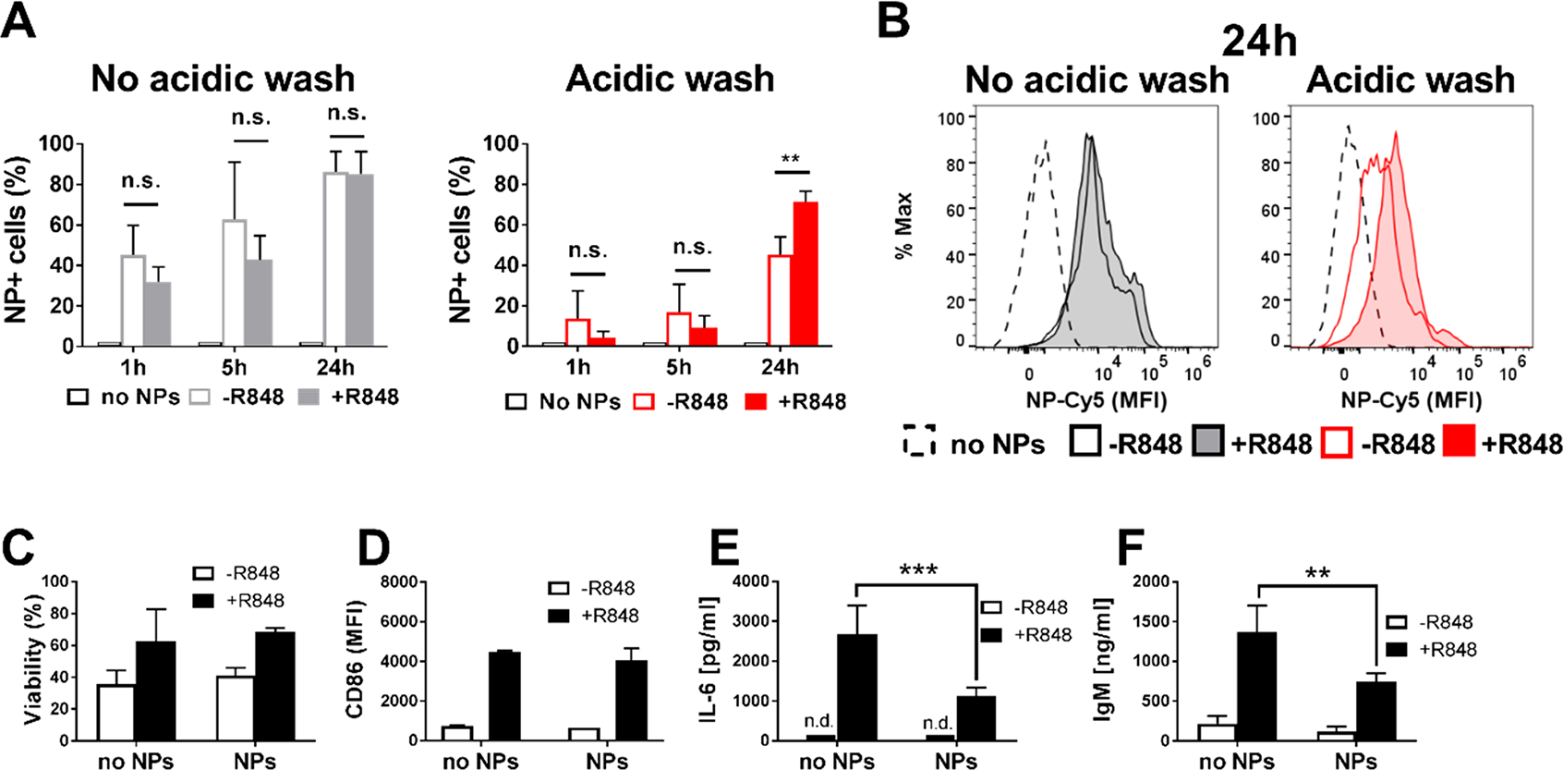
GNP effect on isolated splenic B cells *in vitro*. (A) Total GNP-B cell association (no acidic wash) or GNP uptake (acidic wash) by isolated splenic B cells 1, 5, and 24 h after exposure to 20 μg/mL GNPs in the presence or absence of R848 (2 μg/mL) adjuvant, measured by flow cytometry as a percentage of GNP-Cy5 positive cells. (B) Representative histograms of median fluorescence intensity (MFI) of GNP-Cy5 associated with B cells (no acidic wash) or taken up by B cells (acidic wash), 24 h postexposure in the presence or absence of R848 adjuvant. (C) B cell viability after 24 h exposure with GNPs measured by flow cytometry using amine-reactive viability dye. (D) Expression of surface activation marker CD86 on B cells, measured by flow cytometry 24 h after exposure. (E) IL-6 production by B cells measured in the supernatant by ELISA 24 h after exposure with GNPs. n.d.: not detected. (F) Production of IgM measured in the supernatant by ELISA 24 h after exposure to GNPs. All data show three separate experiments combined (*n* = 3). Error bars of all bar plots: mean ± SD of the pooled data. All data (A–F) were evaluated by two-way ANOVA followed by Tukey’s multiple comparison post hoc test. n.s.: not significant = *p* > 0.05, **p < 0.01, ***p < 0.001. Gating strategy, see Figure S10.

To study the impact of GNPs on activated B cells, we treated them with the TLR7 ligand R848 (resiquimod) a well-known B cell stimulant. In fact, B cells exposed to R848 upregulate activation markers and secrete cytokines such as IL-6.^28^ Isolated B cells were treated with R848 (2 μg/mL) and exposed to a high concentration of GNPs (20 μg/mL) for 1, 5, or 24 h at 37 °C, 5% CO_2_. The results showed that GNP-B cell association (without acidic wash) increased equally in R848-treated or untreated cells in a time-dependent fashion. Similarly, GNPs uptake (with acidic wash) increases with time. However, after 24 h GNP internalization was significantly higher in R848-activated B cells than in resting B cells (Figure 3A). Moreover, the fluorescence intensity of Cy5-labeled GNPs is also increased in R848-treated cells, indicating that they take up more GNPs in comparison to untreated cells (Figure 3B). Taken together, these data suggest that TLR7 stimulation enables more cells to take up GNPs and, at the same time, induces a higher internalization rate in B cells. Moreover, upon incubation of B cells at 4 °C, no GNP-B cell association or uptake was observed, suggesting that GNP association with B cells requires an active metabolic process that is greatly reduced at 4 °C (Figure S4E and S4F). Despite an increased uptake of GNPs by activated B cells, cell viability and expression of CD86 were not affected by GNP exposure in R848-activated B cells (Figure 3C and 3D). However, R848-induced IL-6 levels and total IgM secretion decreased in B cell supernatants after 24 h of exposure to GNP (Figure 3E and 3F). Additionally, a different GNP formulation, without the second layer of PEG (homofunctionalized GNPs; Figure S4G) was tested on isolated splenic B cells showing highly comparable results, suggesting that the incorporation of the Cy5 fluorochrome is stable and does not contribute to GNP–B cell interactions in vitro (Figure S4G–O). Taken together, these results suggest that polymer-coated GNPs at high concentrations inhibit the innate immune function of mouse B cells *in vitro*.

### GNPs Do Not Affect Function of Adjuvant-Activated B Cells in Mice

**Figure 4:**
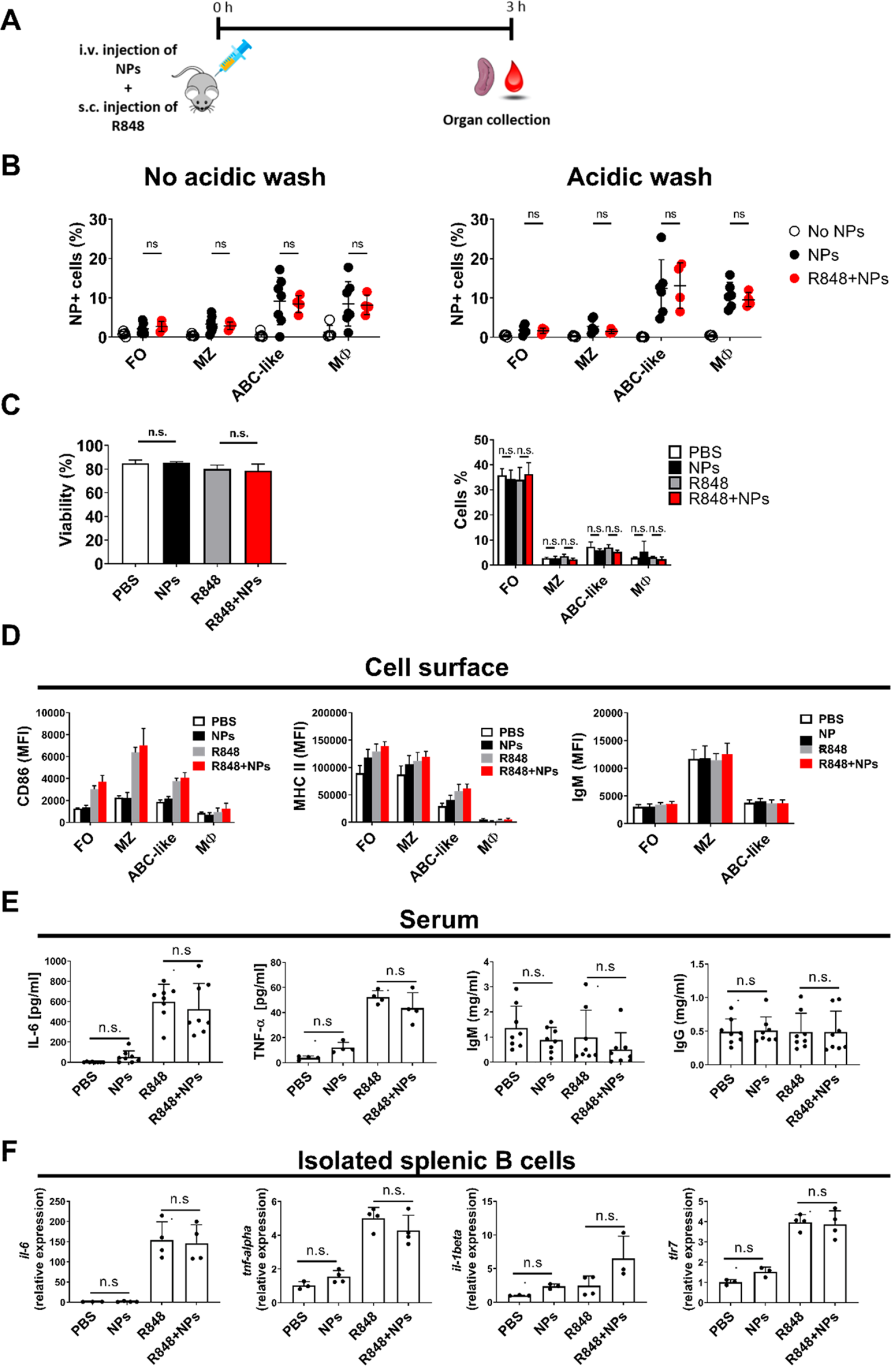
GNP uptake and cell viability after simultaneous injection of GNPs and adjuvant. (A) Scheme of the experimental setup. C57/BL6 mice were intravenously injected with 400 μg GNPs, immediately followed by sub-cutaneous injection of 10 μg R848. Spleen and blood were collected after 3 h. (B) Total GNP-cell association (no acidic wash) and uptake of GNPs (acidic wash) measured in different immune cell populations in the spleen. Each dot shown in B represents an individual mouse (*n* = 4–5). (C) Viability of total splenocytes expressed as percentage of live cells (right panel) and percentage of B cell subsets and macrophages measured by flow cytometry (left panel). (D) Expression of CD86, MHC II, and IgM surface markers in splenic B cell subsets, measured by flow cytometry. MZ: marginal zone B cells (CD19^+^/CD21^+^/CD23^–^), FO: follicular B cells (CD19^+^/CD21^+^/CD23^+^), ABC-like B cells (CD19^+^/CD21^–^/CD23^–^), Mφ (CD11b^+^/CD11c^–^). (E) Production of serum IL-6, IgM, and IgG measured by ELISA. Each dot represents one mouse (*n* = 4–8). (F) Expression of *Il-6*, *Tnf*, *Tlr7*, and *Il-1β* genes in isolated B cell populations measured by qRT-PCR. Data are presented as a relative expression with respect to *Nadph* housekeeping gene. Data shown represent one (F), one of two (C), and two pooled (B,D,E) independent experiments. Significance was evaluated by one-way (C right panels, E, F) and two-way (B, C left panel, D) ANOVA followed by Tukey’s multiple comparisons post hoc test. n.s.: not significant = *p* > 0.05. Error bars in all bar plots: mean ± SD. Gating strategy, see Figure S8.

Based on the results obtained in isolated mouse B cells, the uptake of GNPs by activated B cells *in vivo* was assessed. In order to induce innate-like immune activation of B cells, mice were injected with R848 as previously described.^39^ GNPs (400 μg, i.v.) and R848 (10 μg, s.c.) were injected simultaneously (Figure 4A). In this experimental setup, no difference was observed in GNP uptake by macrophages and B cells between R848-treated and untreated mice at 3 h postinjection (Figure 4B). Importantly, GNPs did not cause a decrease in splenocyte viability or a change in B cell population percentages in adjuvant-stimulated mice (Figure 4C). Furthermore, GNPs did not interfere with the R848-induced upregulation of the B cell activation markers (CD86, MHC II, surface IgM) at 3 h after stimulation (Figure 4D), nor did they impact the concentration of pro-inflammatory cytokines in the serum (IL-6 and TNF-α), as well as total serum IgM and IgG (Figure 4E). Similarly, the transcriptional levels of *Il6*, *Tnf*, *Tlr7*, and *Il1b* in purified B cells collected form R848-treated or untreated mice were not influenced by simultaneous injection of GNPs (Figure 4F). Overall, these results showed that the early B cell innate immune responses were not affected by GNPs in adjuvant-stimulated mice.

### Polymer-Coated GNPs Associate with ABC-like B Cells and Plasmablasts in OVA-Immunized Mice

**Figure 5:**
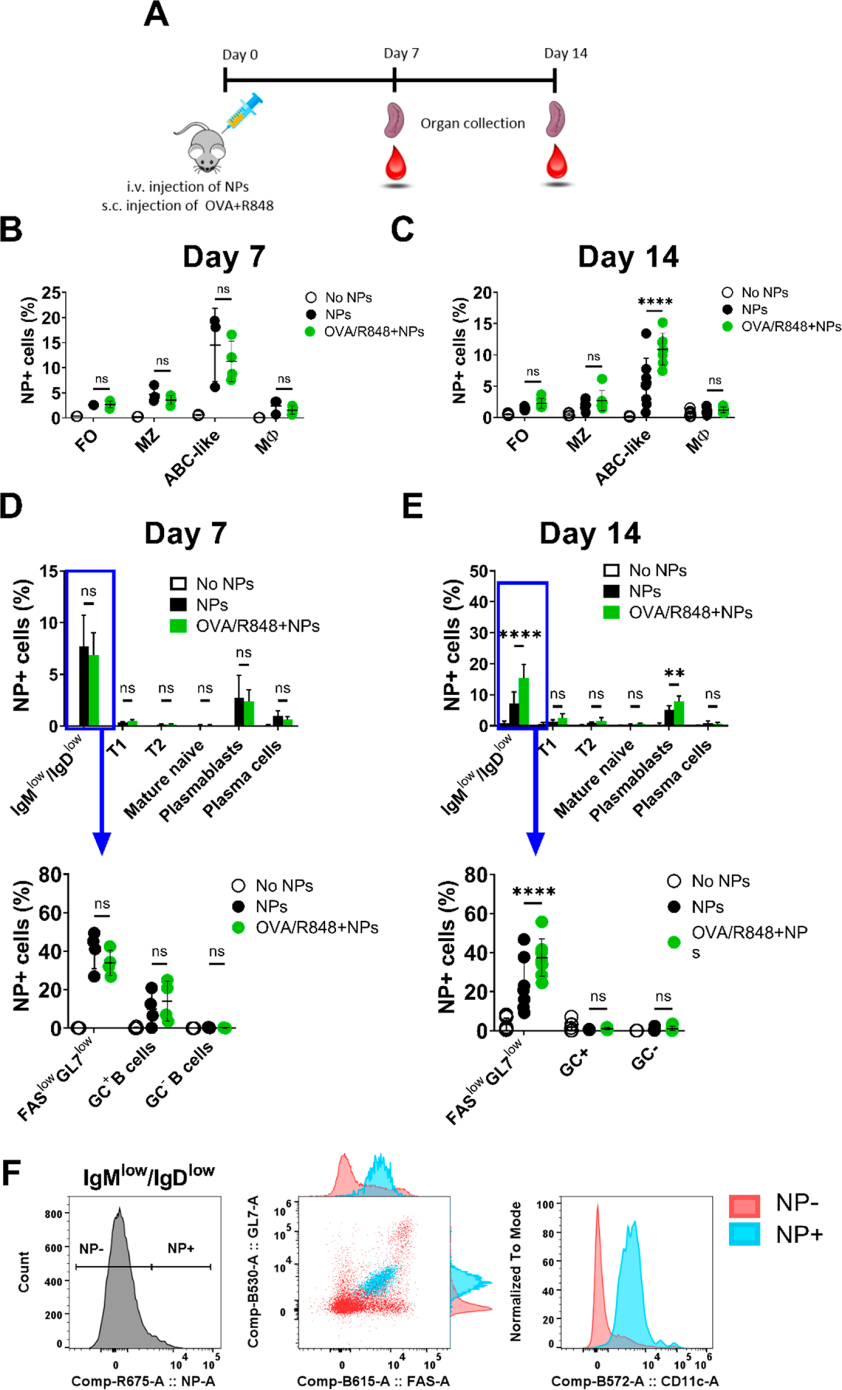
GNP association with different B cell subsets in OVA-immunized mice. (A) Scheme of the experimental setup. C57/BL6 mice were injected intravenously with 400 μg GNPs, immediately followed by injection of a mixture of OVA antigen (100 μg) and R848 adjuvant (10 μg). GNP-cell association was measured by flow cytometry as percentage of GNP-Cy5 positive cells. GNP association measured in different subsets of B cells and macrophages after (B) 7 days or (C) 14 days. MZ: marginal zone B cells (CD19^+^/CD21^+^/CD23^–^), FO: follicular B cells (CD19^+^/CD21^+^/CD23^+^), ABC-like B cells (CD19^+^/CD21^–^/CD23^–^), Mφ (CD11b^+^/CD11c^–^). (D,E) GNP-cell association in different B cell subsets (D) 7 days and (E) 14 days after injection. T1: transitional 1 (CD19^+^/IgM^hi^/IgD^low^), T2: transitional 2 (CD19^+^/IgM^hi^/IgD^hi^), mature naive B cells (CD19^+^/IgM^low^/IgD^int^), plasmablasts (CD19^+^/CD138^+^), plasma cells (CD19^–^/CD138^+^), GC: germinal center B cells (CD19^+^/IgM^low^/IgD^low^/GL7^+^/Fas^+^), GC negative B cells (CD19^+^/IgM^low^/IgD^low^/GL7^–^/Fas^–^), Fas^low^GL7^low^ B cells (CD19+/ IgM^low^/IgD^low^/GL7^low^/Fas^low^). (F) Representative plots of GNP-associated B cells gated on CD19^+^/IgM^low^/IgD^low^. Panel on the left shows the gating of GNP-positive (NP+) and negative (NP−) cells. Middle panel and right panel show dot plots and histograms of GL7, FAS, and CD11c expression on NP+ cells (in light blue) and NP- cells (in light red). Data (B–E) show one (Day 7) and two pooled experiments (Day 14) and were evaluated by two-way ANOVA followed by Tukey’s multiple comparison post hoc test. Each dot represents one mouse (*n* = 4–8). Error bars: mean ± SD; **p < 0.01, ****p < 0.0001, n.s.: not significant = *p* > 0.05. Gating strategy, see Figure S8 and S11.

B cells play a crucial role in adaptive immunity, as they are the sole producers of antibodies upon the encounter with an antigen. Therefore, it is instrumental to investigate the impact of GNPs on B cells adaptive functions. Typically, immunized mice produce antigen-specific IgM approximately 1 to 2 weeks after immunization. Germinal centers form upon encounter of mature B cells with an antigen, inducing a rapid B cell clonal expansion, antibody class-switch, and somatic hypermutations of the antibody variable genes, leading to the development of high affinity antigen-specific antibodies 3 weeks post-primary immunization.^40,41^ To test the effect of GNPs in this context, mice intravenously injected with 400 μg of GNPs were simultaneously immunized with chicken ovalbumin (OVA) and R848. After 7 or 14 days, spleen and blood were collected and GNP–immune cells association was examined (Figure 5A). On day 7, GNPs associated equally in FO, MZ, and ABC-like B cells of OVA-immunized mice and non-immunized mice (Figure 5B), whereas on day 14, association of GNPs with ABC-like B cells was higher in immunized mice (∼10%) than in non-immunized mice (∼5%), suggesting that adjuvant-activated ABC-like B cells have prolonged association with GNPs (Figure 5C). This observation is in line with our *in vitro* data (Figure 3), where R848-stimulated cells internalized more GNPs, and with literature as ABCs are known to be activated and proliferate in response to TLR7 or TLR9 stimulation.^19,20^

Next, B cells were further analyzed according to their differentiation status, which is classified as follows: transitional 1, transitional 2, mature naive B cells, plasmablasts, and plasma cells. CD19^+^/IgM^low^/IgD^low^ cells were further divided into germinal center B cells (GL7^+^/Fas^+^), an intermediate population of GL7^low^/FAS^low^ B cells, and non-GC B cells (GL7^–/^Fas^–^).^42,43^ Flow cytometry analysis revealed that GNPs mainly associated with GL7^low^/FAS^low^ B cells and plasmablasts on day 7, regardless of immunization (Figure 5D). Again, on day 14 GNPs kept higher association with GL7^low^/FAS^low^ B cells and plasmablasts in immunized mice than in control mice (Figure 5E). Considering that CD23^–^/CD21^–^ and GL7^low^/FAS^low^ B cells follow mirroring dynamics of association with GNPs (Figure 5), we confirmed that they constitute overlapping populations of ABC-like cells by FACS analysis (data not shown). In line with this, we observed that the great majority of GNP-positive IgM^low^/IgD^low^ B cells express intermediate levels of GL7 and FAS and are CD11c-positive (Figure 5F). These data are in line with the phenotype of ABC cells which, along with the previously mentioned markers, are characterized by expression of FAS,^44^ and low expression of IgM and IgD.^20^

### Polymer-Coated GNPs Do Not Impact the Viability of B Cell Subsets in Immunized Mice

Prolonged accumulation of inorganic GNPs in the body may have a delayed effect on immune cells, which can result in a change in lymphocyte populations.^8^ The impact of GNPs on the overall viability of splenocytes and on the relative proportions of different B cell subsets was assessed at 7 and 14 days postinjection (Figure S5). Importantly, the results showed no adverse effect of GNPs on splenocyte viability or macroscopic changes in B cell follicle architecture after 14 days (Figure S6). Similarly, GNPs did not cause a change in B cell sub-populations, irrespective of immunization with OVA (Figure S5B, S5C, and S5D). As expected, administration of OVA and R848, regardless to GNPs injection, decreased the percentage of mature naïve B cells and favored the increase of IgM^low^IgD^low^ and FAS^low^GL7^low^ populations (Figure S5C and S5D). This observation may partially explain the higher percentage of GNP-positive cells detected in immunized mice, as ABC-like cells expand upon TLR7 activation.^20,45^ Moreover, no GNP-dependent change of surface activation markers (CD86, MHC II, and IgM) was observed in MZ, FO, and ABC-like B cells (Figure S7). Accordingly, the production of OVA-specific IgM antibodies measured in the serum was unvaried both at day 7 (Figure S7C) and at day 14 in immunized mice exposed to GNPs (Figure S7D). Thus, GNPs do not interfere with B cell antigen-specific immune response *in vivo* and remain B-cell biocompatible after longer exposure in non-immunized as well as immunized mice.

### GNP-Positive B Cells Have an ABC-like Expression Profile and Express Markers of Phagocytic Cells

**Figure 6:**
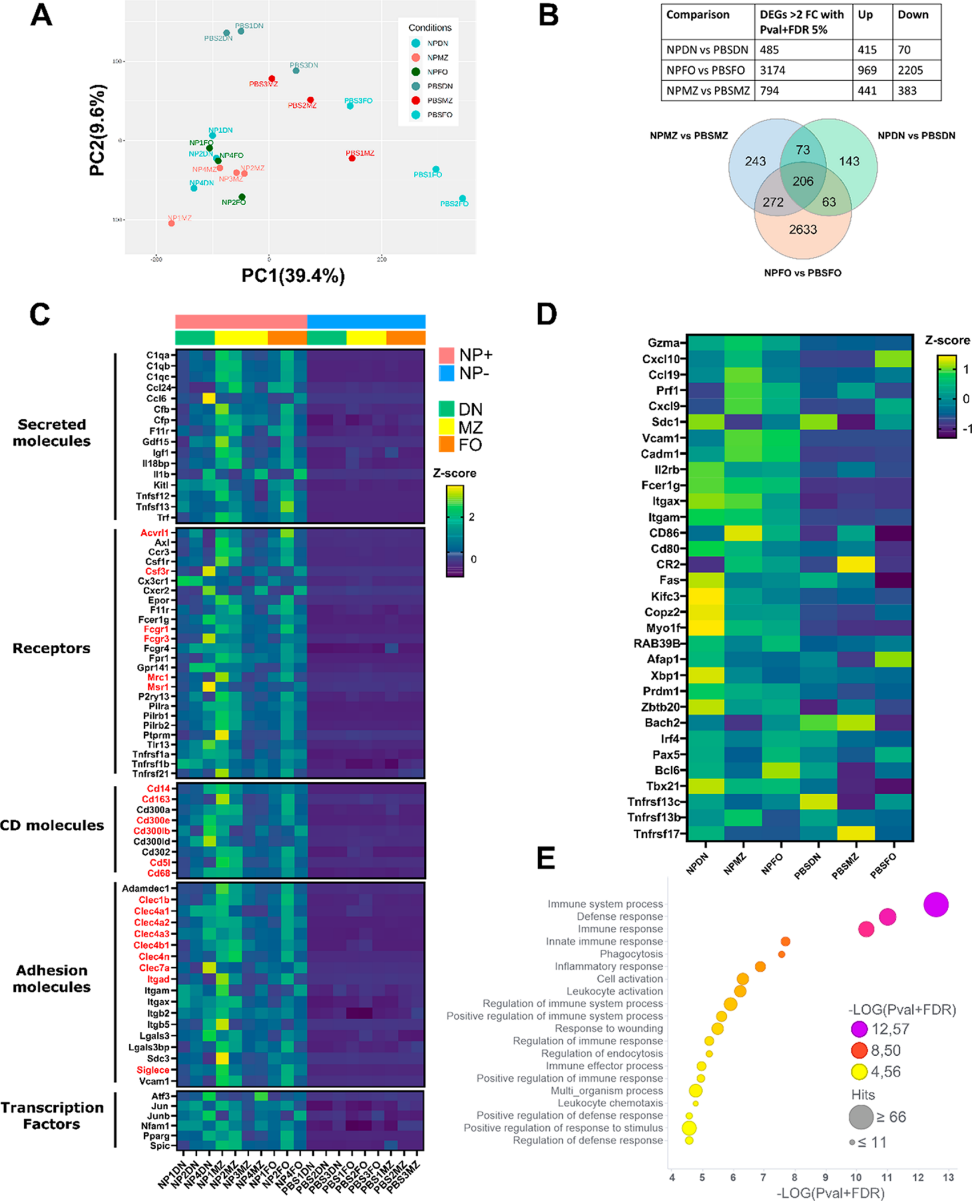
Transcriptional analysis of GNP-associated B cell sub-sets. C57/BL6 mice were injected with 400 μg GNPs and OVA (100 μg)/R848 (10 μg) or PBS and after 14 days. GNP-positive (NP^+^) cells from the B cell subsets and control (PBS) cells were sorted according to CD21 and CD23 expression for RNAseq analysis. FO B cells: CD19^+^/CD3^–^/CD21^+^/CD23^+^, MZ B cells: CD19^+^/CD3^–^/CD21^+^/CD23^–^, and ABC-like double negative (DN): CD19^+^/CD3^–^/CD21^–^/CD23^–^. (A) Principal component analysis (PCA) of GNP-associated and control B cells (*n* = 3–4). (B) Table of 2-fold significantly (*p*-value > 0.05 + 5% FDR) differentially expressed genes (DEGs) between GNP^+^ and control cells (top panel), and Venn diagram showing common DEGs (bottom panel). NPMZ: GNP^+^ MZ B cells, NPFO: GNP^+^ FO B cells, NPDN: GNP^+^ ABC-like B cells, PBSMZ: PBS control MZ B cells, PBSFO: PBS control FO B cells, and PBSDN: PBS control ABC-like B cells. (C) Selection of common DEGs between GNP^+^ and PBS control B cells. Myeloid cell genes highlighted in red. (D) GNP^+^ B cells express signature genes of ABC B cells. (E) Overall-representation analysis (ORA) of top 20 GO biological processes (BP) pathways. Symbol color indicates FDR-corrected *p*-value of the enrichment analysis. Symbol dimensions indicate the number of hit genes.

To better understand the impact of GNP association with the different B cell sub-sets, we sorted GNP-positive B cells from the spleen and performed gene expression analysis by RNAseq. Interestingly, we observed that GNP^+^ B cells from the FO, MZ, and ABC-like (CD21/CD23 double negative cells, DN) sub-sets showed similar transcriptional profiles, as they group together after principal component analysis (PCA), in opposition to FO and MZ B cells isolated from PBS-treated mice, which formed distinct clusters (Figure 6A). Combining the DEGs from the three subsets, we identified a pool of 206 common DEGs in NP^+^ B cells (Figure 6B). Among these, genes encoding several immune-relevant secreted mediators of the complement and coagulation cascade (*C1qa*, *C1qb*, *C1qc*, *Cfb*, *Cfp*, *F11r*), pro-inflammatory molecules (*Il1b*, *Ccl6*, *Ccl24*), and the B cell survival factor APRIL (*Tnfsf13*) (Figure 6C) were detected. Interestingly, together with the typical hallmarks of ABC B cells CD11b and CD11c (*Itgam* and *Itgax*), we observed upregulation of numerous other receptors and surface molecule genes usually expressed in myeloid cells (Figure 6C, highlighted in red). Notably, the CD21^–^CD23^–^ (double negative, DN) cells express many ABC-specific signature genes^20^ including the transcription factor T-bet (*Tbx21*), confirming our hypothesis that they are ABC B cells (Figure 6D). This gene signature was increased in GNP^+^ cells of all the sub-sets, suggesting some degree of plasticity between the MZ and FO cells and ABC cells. In line with this, it has been shown that ABC can derive from FO B cells.^19,45,46^ Moreover, ABCs include specific sub-sets of antigen-experienced^47,48^ memory B cells^49^ primed toward the plasma cell lineage.^50,51^ This concept is confirmed by the fact that GNP^+^ cells (in particular of the DN subset) highly expressed a profile typical of plasma cell differentiation including upregulation of the transcription factors *Prdm1* (Blimp1), *Irf4*, *Zbtb20*, and *Xbp1*, and downregulation of *Bach2*. Nonetheless, they still expressed *Bcl6*, which is typically downregulated in plasma cells, indicating that these cells did not fully complete their differentiation to antibody-secreting plasma cells. This is in line with literature suggesting that ABCs may constitute a memory B cell pool of plasma cell precursors.^21,51^

Considering the upregulation of several scavenger receptors and phagocytic receptors on GNP^+^ B cells, we speculated that ABC-like B cells might have enhanced phagocytic capacities, which would explain their superior ability to associate with GNPs. This hypothesis was supported by the observation that phagocytosis is one of the top enriched Gene Ontology (GO) biological pathways (BP) among the common DEGs (Figure 6E). Our data are in line with literature, as ABCs have previously been shown to have superior antigen-presenting abilities in an antigen concentration-dependent manner, suggesting a higher phagocytic potential.^52^

## Conclusions

Understanding the interactions between GNPs and B cells and the impact of GNPs on B cell function is crucial for the future design of medically applicable GNPs. This is especially important in GNP-based vaccine applications, where B cells are often directly targeted. Our findings reported here provide insights about the general biocompatibility of polymer-coated GNPs and their ability to interact with B cells and their primary and adaptive immune responses *in vivo*. Surprisingly, we observed significant selective uptake of GNPs by ABCs, which has never been investigated so far.

Our results suggest that in the first 24 h after systemic administration GNPs interact with MZ B cells and ABC-like B cells. They are also able to reach FO B; however, they are not significantly internalized by this subset at this time point. A similar observation was reported before, where 50 nm PEGylated GNPs were predominantly visualized in the marginal zone and red pulp after 1 h, followed by broader distribution and deeper detection in the follicles after 24 h.^53^ The early association of GNPs with MZ B cells may be explained by the possible opsonization of GNPs with serum proteins such as complement proteins and the ability of B cells to phagocytose opsonized particular matter *via* complement receptors (e.g., C1R), which are highly expressed on MZ B cells.^54^ Moreover, their localization to the marginal zone offers a privileged access to the circulation and blood-borne antigens favoring possible interactions with GNPs. Similarly, ABCs are localized between the T cell and B cell borders and in the T cell zone constituting the periarterioral lymphoid sheaths (PALS).^22^ Moreover, a recent publication located CD11c^+^ Tbet^+^ B cells in the marginal zone.^35^ The authors demonstrated that a significant fraction of CD11c^+^ Tbet^+^ B cells is labeled upon i.v. injection of fluorescently conjugated antimouse CD45 Ab, which confirms their localization within compartments exposed to open blood circulation.^35^ The proximity of ABCs to blood vessels may explain the kinetics of their association to GNPs, which can happen as early as 3 h after intravenous or sub-cutaneous injection. Additionally, we could not observe any strong association of GNPs with B cells in the liver, blood, and lymph nodes. However, Tsoi et al. reported high uptake of PEGylated quantum dots (*d*_*h*_: ∼15 nm) by hepatic B cells 4 and 12 h after systemic administration.^55^ This suggests that different B cell subtypes have different phagocytic ability and specificity, which results in the uptake of nanoparticles depending on their physiochemical characteristics.

The exposure of primary murine B cells *in vitro* to high concentrations of GNPs (20 μg/mL) did not cause changes in cell viability nor did it interfere with CD86 expression. However, we observed a significant decrease in IL-6 and IgM production in TLR7-stimulated B cells. In our previous study on primary human B cells, we observed comparable results with the same GNP exposure concentrations.^28^ However, rod shaped PEGylated GNPs and polymer-unprotected citrate-stabilized gold nanospheres caused similar suppression of IL-6 production in TLR7-stimulated B cells.^28^ These results together suggest that GNPs have the ability to attenuate human and mice B cell innate immune response under certain circumstances. The inconsistency between the suppressive effect observed *in vitro* and the lack of it *in vivo* might be caused by a possible inability of GNPs to reach B cells in a sufficient dose to cause suppression in the tested conditions. Importantly, we detected higher frequencies and increased uptake in GNP-internalizing B cells upon TLR stimulation. Similar results were reported by Dutt et al. where higher uptake of single-walled carbon nanotubes in LPS-activated B cells caused an increase in B cell toxicity *in vivo*.^56^ In contrast, we observed no decrease in cell viability or modulation of B cell early immune response in our experimental settings. Together, these data suggest high biocompatibility of PEG-coated GNPs with B cells *in vivo*. However, the long-term effects of GNP exposure remain to be clarified, especially regarding their impact on B cell functions.

Several studies demonstrated that ABCs are involved in multiple aspects of adaptive immunity including infectious diseases, vaccination,^50,57−59^ and autoimmunity.^20,60−63^ Moreover, they contribute to humoral immunity by producing antigen-specific antibodies, either against microbes^35,64,65^ or self-molecules,^20,66,67^ indicating that they could play beneficial or detrimental roles depending on the context. Additionally, ABCs participate in the process of immunosenescence as they accumulate with age, occupying the same niche as FO B cells, suggesting that ABCs play more prominent roles over time.^21^ Our data demonstrate that GNPs may be used as a tool to specifically target ABCs due to their unique association properties, which highlights future possible research focuses and clinical applications of GNPs.

One possibility would be to exploit the superior antigen-presenting capacities^52^ of ABCs to produce more effective vaccines in the elderly, where ABCs constitute a significant fraction of splenic B cells and traditional vaccines are less efficacious. Considering that B cells can take up antigen-coated NPs through BCR-dependent phagocytosis, which leads to efficient formation of B cell germinal centers,^7^ it is possible to consider GNPs as vehicle for targeted delivery of antigens to ABCs. Alternatively, GNPs could also be used to target ABCs in autoimmune diseases. GNPs may be functionalized with ABC-suppressive drugs in order to limit their expansion and the production of autoantibodies. In this indication, a recent study showed that activation of the adenosine receptor 2A using the agonist CGS-21680 results in the depletion of CD11c^+^ Tbet^+^ B cells in both *E. muris*-infected and lupus-prone mice,^68^ making it a strong candidate for targeted delivery using GNPs.

The observation that only a certain percentage of ABC-like B cells take up GNPs suggests that they might represent a particular subset of ABCs with increased phagocytic abilities, which is in line with the heterogeneous nature of ABCs.^21^ In this regard, GNP^+^ ABCs might represent a transitional stage of differentiation that can arise from both FO and MZ cells. However, this fascinating hypothesis, as well as the study of ABC plasticity, needs further investigation in order to better understand the nature of ABC cells and their functions.

In conclusion we demonstrated that polymer-coated GNPs are well tolerated and do not affect the innate or adaptive immune response *in vivo*. Based on our biodistribution data, we showed that GNPs associate with different splenic B cell subpopulations and constitute a promising tool to selectively target ABCs.

## Experimental Methods

### GNP Synthesis and Characterization

Fifteen nanometer citrate-stabilized gold nanospheres were synthesized as reported by Turkevich et. al.^69^ Thiolated PEG polymers (NH_2_-PEG-SH) conjugated with Cy5 fluorochrome were synthesized, followed by polymer coating of GNPs to obtain homofunctionalized fluorescently labeled GNPs as previously reported by Rodriguez-Lorenzo et al.^27^ To obtain hetero-GNP formulation, homofunctionalized GNPs were coated with the second PEG coating (methoxy PEG).^27^

GNP stability was evaluated by the optical characterization with Cary 60 UV–vis spectrophotometer (Agilent Technologies). Prior to measurements of GNP spectra, 20 μg/mL of GNPs were incubated in water, PBS, complete culture medium (RPMI 1640 with l-Glutamine culture medium (Gibco) supplemented with 10% fetal calf serum (FCS, Biowest), 1% penicillin/streptomycin, 1% sodium pyruvate, 1% nonessential amino acid (NEAA), 0.1% β-mercaptoethanol (all from Gibco) and 15% mouse serum (obtained from mouse blood) and incubated at 37 °C overnight. The size and ζ-potential of GNPs were measured with Zetasizer 7.11 (Malvern) using dynamic light scattering (DLS) and electrophoretic light scattering, respectively. For this purpose, GNPs were diluted in water to 0.05 mg/mL.

### Mice

7–12 weeks old female C57BL/6J mice were used throughout the experiments. Mice were maintained under specific pathogen-free conditions in the animal facility at the Centre Médical Universitaire, University of Geneva. All animal experiments were conducted according to Swiss ethical regulations (license: GE_93_19, GE_182_19 and GE_93_20). Animals were purchased from Charles River Laboratories, Saint Germain Nuelles, France.

### Organ Processing

Spleens harvested from mice were cut and smashed through 40 μm strainer and washed with PBS. After centrifugation (5 min, 400*g*, 4 °C) supernatant was discarded and red blood cells were removed by adding 1 mL of lysing buffer BD Pharm Lyse (BD Biosciences) for 1 min at RT. Lymph nodes were cut and incubated at 37 °C on a shaker (150 rpm) for 20–30 min in complete culture medium with 2 mM CaCl2 (Acros Organics), 3 mg/mL collagenase and 200 U/mL DNase I (both from Worthington). After incubation, digested lymph nodes were filtered through 40 μm strainer and washed with PBS. Liver tissue (∼750–1200 mg) was dissociated with a liver dissociation Kit (Miltenyi), following the manufacturer’s protocol, and further processed with gentle MACS Dissociator with Heaters run with the 37C_m_LIDK_1 program (Miltenyi). After obtaining a cell suspension from liver tissue, red blood lysis was performed (1 min, RT). ∼100 μL of the collected blood sample was added into PBS with 2% EDTA, followed by red blood lysis (5 min, RT) and several washes with PBS, prior to flow cytometry measurements. The rest of the blood was used to collect serum by centrifugation with microcentrifuge (30 min, 21 130*g* at RT) and stored at −20 °C for further ELISA assay.

### GNP Exposures *In Vitro*

Heterogeneous cell suspensions from spleen or LN were exposed to GNPs (20 μg/mL) for 24 h at 37 °C, 5% CO_2_. R848-ALX (Enzo) immunostimulant (2 μg/mL) was used as a positive control for activation. After GNP exposure, supernatants were collected and stored at −20 °C for ELISA analysis. Isolated splenic B cells were exposed to different formulations (homo and hetero) of GNPs for 1, 5, and 24 h at 37 °C and for 1 and 5 h at 4 °C at GNP concentration of 20 μg/mL. R848 immunostimulant (2 μg/mL) was used as a positive control for activation of innate-like B cell immune response.

### GNP Biodistribution

Mice were injected intravenously with 400 μg GNPs in the tail vein or with 300 μg GNPs subcutaneously in the flank. Three h or 24 h after injections mice were euthanized by CO_2_ inhalation and different organs (spleen, liver, LN) and blood were collected and processed as described above. Acidic wash was then performed on cell suspensions of the spleen, LN, liver, and blood, followed by staining for flow cytometry measurements.

### Effect of GNPs in Adjuvant- and Antigen-Stimulated Mice

Mice were intravenously injected with 400 μg GNPs in PBS. Immediately after, a mixture of 100 μg ovalbumin (OVA, Invivogen) and 10 μg R848 in PBS was subcutaneously injected into mouse flank. On day 7 or day 14, mice were euthanized by CO_2_ inhalation and spleen and blood were collected and processed for further flow cytometry and ELISA analysis, respectively.

### Flow Cytometry and Cell Sorting

NovoCyte 3000 (ACEA Biosciences) was used for all flow cytometry measurements. Prior to flow cytometry measurements, compensation for the corresponding staining was performed by compensation beads UltraComp eBeads (Invitrogen). FlowJo (version 10) was used for postmeasurement analysis. Cells were first stained with Zombie viability dye (Biolegend) in 1:1000 dilution with PBS and incubated for 15 min at RT. Next, cells were washed with FACS buffer (0.5% BSA and 0.5 mM EDTA in PBS). Cells were then stained with the corresponding antibody mix diluted (all diluted at 1:200 in FACS buffer unless stated otherwise). FcBlock TruStain fcX-CD16/32 (1:100, Biolegend) was added into the mix. Gating strategies for in vitro and in vivo experiments are presented in Figures S8, S9, S10, and S11. Cell sorting was performed after immunostaining described above using the FACSAria Fusion (BD Biosciences) or the MoFlo Astrios EQ (Beckman Coulter) cell sorters according to the gating strategies described in Figure S12. After sorting cells were lysed in TRIzol (Thermo Fisher) and stored at −20 °C.

### Quantitative Real-Time PCR (qRT-PCR)

Mice were simultaneously injected with 400 μg GNP (i.v.) and 10 μg R848 (s.c.). After 3 h of incubation, mice were euthanized by CO_2_ inhalation and pure splenic B cell population was isolated with mouse B cell Isolation Kit (negative selection, Miltenyi Biotech), following the manufacturer’s protocol. Next, RNA was isolated from pure B cells by trizol-based RNA extraction as described in supporting methods. RNA concentration was measured by NanoDrop ONE^C^ (Thermo Scientific). One μg of RNA was used for cDNA synthesized by High-Capacity cDNA Reverse Transcription Kit (Applied Biosystems), following the manufacturer’s instructions. RNA expression was quantified by QuantStudio 5 qRT-PCR machine (Applied Biosystems) using PowerUP SYBER Green Master Mix (Applied Biosystems). The following primers were used: housekeeping gene *Gapdh*: CAAAGTGGAGATTGTTGCCA (forward), GCCTTGACTGTGCCGTTGAA (reverse); *Tlr7*: TGATCCTGGCCTATCTCTGAC (forward), CGTGTCCACATCGAAAACA (reverse); *Il-6*: AGTCCGGAGAGGAGACTTCA (forward), ATTTCCACGATTTCCCAGAG (reverse); *Tnf-alpha*: AAATGGCCTCCCTCTCAT (forward), CCTCCACTTGGTGGTTTG (reverse); *Il-1beta*: GAAGAAGAGCCCATCCTCTG (forward), TCATCTCGGAGCCTGTAGTG (reverse).

### RNAseq

RNA was extracted from FACS-sorted B cells with the RNA Clean and Concentrator-5 kit (Zymo research) following manufacturer’s instructions. RNA concentration was measured with a Qubit fluorimeter (Life Technologies) and RNA integrity assessed with a Bioanalyzer (Agilent Technologies). The SMART-Seq v4 Ultra Low input RNA kit (Clontech Laboratories, Inc.) followed by the Nextera XT DNA library Prep kit (Illumina) were used for the library preparation. Library molarity and quality were assessed with the Qubit and Tapestation using a DNA High sensitivity chip (Agilent Technologies). The libraries were pooled at 2 nM and loaded for clustering on lane of a Single-read Illumina Flow cell. Reads of 100 bases were generated using the TruSeq SBS chemistry on an Illumina HiSeq 4000 sequencer. The normalization and differential expression analysis was performed with the R/Bioconductor package EdgeR, for the genes annotated in the reference genome (http://www.ncbi.nlm.nih.gov/pmc/articles/PMC2796818/). Counts were filtered and normalized to library size (sequencing depth) and RNA composition (dispersion). Each sample is scaled using the TMM (Trimmed Mean of M-values) normalization method. Pairwise comparison of the libraries and calculation of differentially expressed genes was performed using the R/Bioconductor package EdgeR generalized linear model and quasilikelihood F-test. PCA analysis and Overrepresentation Analysis (ORA) were produced with the web-based bioinformatics package NetworkAnalyst (http://www.networkanalyst.ca) based on recommended protocol. Sequencing data are available on the database repository Gene Expression Omnibus (GEO) (https://www.ncbi.nlm.nih.gov/geo/) with the series accession number: GSE197944. Lists of differentially expressed genes, common DEGs, and GO BP enrichment pathway are reported in Table S1.

### Histology

Mice were euthanized by CO_2_ inhalation and spleens were collected. Pieces of spleens were cut, washed with PBS, and fixed in freshly prepared 4% PFA (pH ∼ 6.9) for 24 h at 4 °C, gently agitated. Next, spleen samples were washed with PBS and placed in 15% sucrose for 3 h, followed by incubation for 6 h in 30% sucrose at 4 °C, gently nutated. Samples were then placed in OCT (VWR Chemicals), snap-frozen on dry ice and stored at −80 °C. Spleen samples embedded in OCT were cut at 5 μm thickness by cryostat (Thermo Fisher) and placed on glass slides. Slides with spleen tissue were stained by hematoxylin and eosin, following the standard protocol.^70^ Images were obtained by slide scanning microscope at ×20 magnification, brightfield (Zeiss Axioscan.Z1) and analyzed with Zeiss 3.0 software.

### ELISA Assays

Mouse IL-6 ELISA MAX Standard Set and ELISA MAX Deluxe Set TNF-α (Biolegend) were used to measure the concentration of cytokines in supernatants or serum, following the manufacturer’s instructions. Total IgG and IgM in supernatants and mouse serum were measured by ELISA, following the in-house developed ELISA protocol described in the Supporting Methods.

### Data and Statistical Analysis

Data were evaluated for significance using unpaired two-tailed Student’s *t* test for comparison between two groups. For comparison between multiple groups, one-way ANOVA, followed by Dunnett’s multicomparison test or two-way ANOVA followed by Tukey’s or Sidak’s multicomparison test was used (GraphPad Prism 7 software). Data were considered significant when *p < 0.05, **p < 0.01, ***p < 0.001, and ****p < 0.0001.

## Supporting Information Available

Supplementary Methods: Primary cell cultures, Acidic wash method (GNP uptake), List of antibodies used in the study, RNA extraction, RNA extraction with Trizol, ELISA IgM and IgG; Supplementary Figures S1–S12 (DOCX)
Supplementary Tables (XLSX)
