# Supplementary material for: PEGylated Gold Nanoparticles Target Age-Associated B Cells In Vivo
Source: ACS Nano. 2022 Oct 27;16(11):18119–32. doi: 10.1021/acsnano.2c04871 (PMC9706664; doi:10.1021/acsnano.2c04871)
Supplement: Supplementary file 1 — nn2c04871_si_001.docx [file nn2c04871_si_001.docx]

Supporting information for

PEGylated gold nanoparticles target Age-Associated B cells *in vivo*

*Sandra Hočevar^1†^, Viola Puddinu^1†^, Laetitia Haeni^3^, Alke Petri-Fink^3^*, *Julia Wagner^1^, Montserrat Alvarez^1^, Martin James David Clift^4#^*, Carole Bourquin^1,2#^*

^1^Institute of Pharmaceutical Sciences of Western Switzerland, University of Geneva, 1211 Geneva, Switzerland

^2^Department of Anaesthesiology, Pharmacology, Intensive Care and Emergency Medicine, Faculty of Medicine, University of Geneva, 1211 Geneva, Switzerland

^3^BioNanomaterials, Adolphe Merkle Institute, University of Fribourg, 1700 Fribourg, Switzerland.

^4^*In Vitro* Toxicology Group, Swansea University Medical School, Swansea, Wales, SA2 8PP, UK.

*^†^* These authors contributed equally as primary author.

^#^These authors contributed equally as senior author.

*Corresponding author (E-mail: [m.j.d.clift@swansea.ac.uk](mailto:m.j.d.clift@swansea.ac.uk))

**Supplementary methods**

**Primary cell cultures**

Obtained heterogeneous cell suspensions from spleen or LN were plated at 2 × 10^5^ cells/well in a U-bottom 96-well plate (Corning) in RPMI 1640 with L-Glutamine culture medium (Gibco) supplemented with 10% fetal calf serum (FCS, Biowest), 1% Penicillin/Streptomycin, 1% Sodium Pyruvate, 1% Non-Essential Amino Acid (NEAA), 0.1% β-mercaptoethanol (all from Gibco). The pure B cell populations were isolated from splenocytes with mouse B cell Isolation Kit (negative selection, Miltenyi Biotech), following the manufacturer's protocol. > 96% purity was reached throughout the experiments. Isolated splenic B cells were plated at 2 × 10^5^ cells/well in a U-bottom 96-well plate in a complete culture medium (as described above).

**Acidic wash method (GNP uptake)**

After GNP exposures, cells were incubated with an acidic wash buffer (100 nM NaCl, 50 nM Glycin in H_2_O; pH 3) for 1 min on ice to remove the cell surface-associated GNPs. Next, cells were washed 2 x with PBS. Cells were then stained as normal with a viability dye and antibodies prior to flow cytometry measurements.

**List of antibodies used in the study**:

*Leukocytes:* CD45-PE-Cy7. *B cells:* CD19-BV510 (6D5), CD19-BV605 (6D5), CD19-BV650 (6D5), CD19-PE-DazzleAF594 (6D5), B220-PerCP (RA3-6B2), CD23-PE (B3B4), CD21/35-PE-Cy7 (7E9), IgM-BV605 (RMM-1), IgD-AF488 (11-26c2a), CD138-PE-Cy7 (281-2), GL7-PE (GL7) GL7-FITC (GL7), CD95 (Fas)-PE-CF594 (Jo2). *T cells:* CD3-FITC (17A2), CD3-Pacific Blue (17A2). *Macrophages and dendritic cells:* CD11b-BV785 (M1/70), CD11b-BV570 (M1/70), CD11c-PE (N418). *Immune activation:* MHC II-FITC (from MACS), CD86-BV510 (GL-1), CD86-BV605 (GL-1), CD86-BV785 (GL-1).

**RNA extraction with Trizol**

Briefly, B cell pellets were collected and lysed with 1 ml of trizol (Life Technologies). Cell lysates were then centrifuged in a microcentrifuge (14000 rpm, 10 min, 4 °C) and the supernatant was collected. 200 ul of biphenol:chloroform:isoamyl alcohol = 25:24:1 (Biosolve) was added and vortexed. Samples were incubated for 3 min at room temperature and centrifuged (12000 rpm, 15 min, 4 °C). Next, the RNA phase was carefully collected and 500 ul of isopropanol was added. Samples were vortexed and incubated for 10 min at RT. After, samples were centrifuged (12000 rpm, 10 min, 4 °C) and the pellet was re-suspended in 1 ml of 75% ethanol. Samples were vortexed and centrifuged for (5 min at 7500 x g, 4 °C). Supernatants were discarded, pellets were dried and re-suspended in 20 ul of nuclease-free water (Invitrogen).

**ELISA IgM and IgG**

Goat-Anti-Mouse IgG and Goat-Anti-Mouse IgM were used as coating antibodies (SouthernBiotech). Unconjugated mouse IgG and mouse IgM isotype control were used for standards (Thermo Fisher). For the detection of IgM and IgG in the samples, Goat-Anti-Mouse Ig-HPR (Southern Biotech) was used. The antibody, standard and sample dilutions used are presented in (Table 1). 96 half-well plates (Corning) were coated with coating antibodies in coating buffer (70 mM NaHCO_3_, 30 mM Na_2_CO_3_ in ddH2O, pH 9.6) and left overnight at 4° C. Next, the plates were washed with 0.05% Tween-20 in PBS and blocked with blocking buffer (2% BSA in PBS) for 2 h at RT. After washing, samples (diluted in ELISA Assay Diluenf from Biolegend) and corresponding standards were added an incubated for 2h at RT. Plates were washed and detection antibodies were added for 30 min at RT. After washing, TMB Substrate Reagent Set (BD Biosciences) was added. After approximately 6 min stop solution (1M H_2_SO_4_) was added. Absorbance was measured at 450 nm and 570 nm by plate reader CLARIOstar (BMG Labtech). For the measurement of OVA-specific IgM antibodies, ELISA was performed, following in-house developed OVA-specific IgM ELISA protocol. 96 half-well plate was coated with 0.5 μg of OVA/well in coating buffer overnight at 4 °C. Next, plates were washed (0.05% Tween-20 in PBS) and blocked (2% BSA in PBS) for 2 h at RT. After washing, serum samples were added and incubated for 2 h at RT. After incubation, plates were washed and detection Goat-Anti-Mouse IgM-HPR antibodies (Southern Biotech) were added. After 1 h of incubation at RT, plates were washed, and substrate (TMB) was added. After sufficient development of the color (~4 min) stop solution (1M H_2_SO_4_) was added. Absorbance was measured at 450 nm and 570 nm by plate reader CLARIOstar (BMG Labtech).

**Table 2:** List of dilutions and concentrations of antibodies, samples or antigens used in the in-house ELISA protocols for total IgM, IgG and OVA-specific IgM.

|  | **IgM** | | **IgG** | | **OVA-specific IgM** |
| --- | --- | --- | --- | --- | --- |
| ***Type of sample*** | *Supernatant* | *Serum* | *Supernatant* | *Serum* | *Serum* |
| ***Coating Ab/Ag*** | 1:1000 | 1:1000 | 1:1000 | 1:1000 | OVA:10 μg/ml |
| ***Samples dilutions*** | 1:4 | 1:10^4^ | 1:4 | 1:10^6^ | 1:100 |
| ***Standards*** | Max at 1000 ng/ml;  1:2 serial dilutions | Max at 200 ng/ml;  1:3 serial dilutions | Max at 200 ng/ml;  1:2 serial dilutions | Max at 200 ng/ml  1:3 serial dilutions | / |
| ***Detection Ab*** | 1:8000 | 1:8000 | 1:8000 | 1:8000 | 1:500 |

**Supplementary Figures**


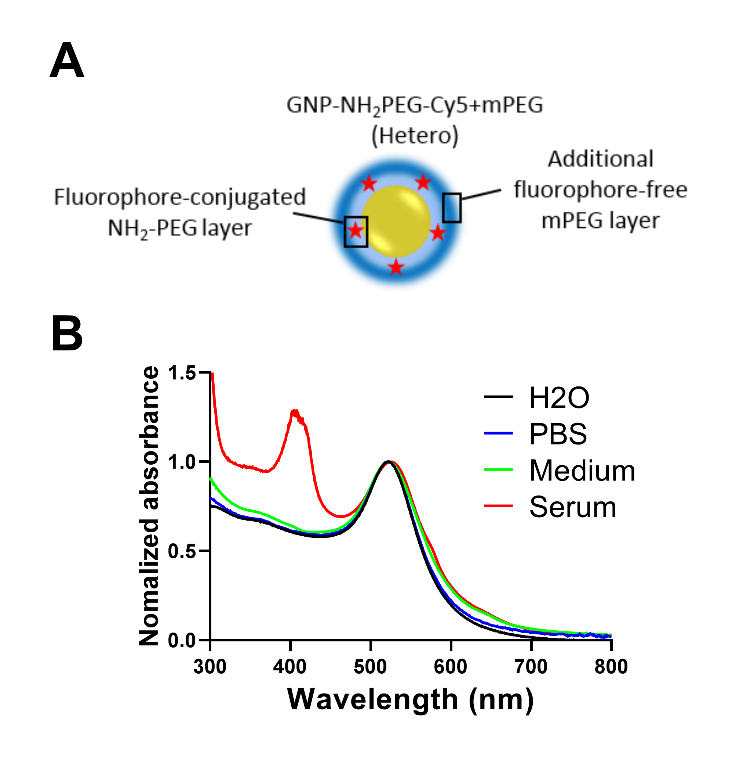
**Figure S1:** Polymer-coated gold nanoparticles and their stability in different biological media. **A)** Schematic presentation of fluorescently-labeled (Cy5) GNPs coated with a double layer of PEG (NH_2_-PEG-mPEG). **B)** UV-Vis spectra of GNPs after incubation in water, PBS, complete RPMI medium with 10% FBS and in 15% mouse serum. The additional peak at ~400 nm for the spectrum of GNP incubated in serum occurred due to the interference of hemoglobin residue. mPEG: methoxy PEG.


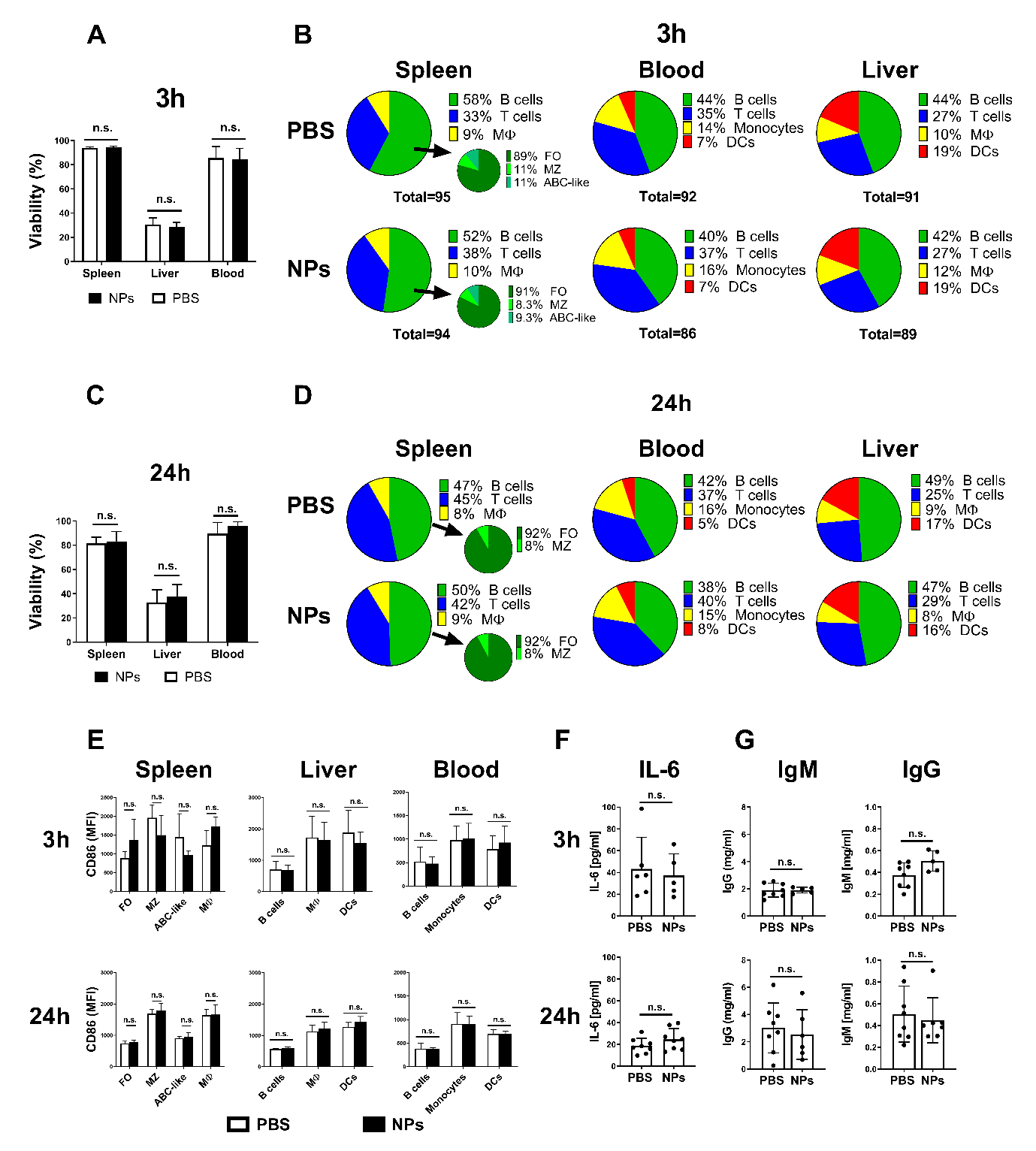


**Figure S2:** Cell viability in different organs after intravenous administration of GNPs. Cell viability (**A** and **C**) and percentage of different leukocyte populations in spleen, liver and blood (**B** and **D**) at 3h and 24h post-intravenous injection with 400 µg GNPs in C57/BL6 mice, measured by flow cytometry using an amine-reactive viability dye. **E)** Expression of the surface immune activation marker CD86 measured by flow cytometry in different immune cell populations of spleen, liver and blood 3 and 24 h post injection with GNPs. Gating strategies shown in Figure S9**:** B cells (CD19^+^/CD3^-^), MZ: marginal zone B cells (CD19^+^/CD21^+^/CD23^-^), FO: follicular B cells (CD19^+^/CD21^+^/CD23^+^), ABC-like B cells (CD19^+^/CD21^-^/CD23^-^), T cells (CD19-/CD3+), Mφ/monocytes (CD11b^+^/CD11c^-^), DC: (CD11b^+/-^/CD11c^+^). Serum concentration of IL-6 **(F)** and total serum IgM and IgG **(G)** measured by ELISA 3 h and 24 h post injection with GNPs. Data for each time point show two separate experiments combined. Each dot represents one mouse (n=5-8). All graphs show pool data of two or three independent experiments (5-12). Error bars in all bar plots: mean ± SD of the pooled data. Data in A, C and E were evaluated by two-way ANOVA, followed by Sidak’s multiple comparison post-hoc test. Data in F and G were evaluated by unpaired Student´s t-test. n.s: not significant = p>0.05.


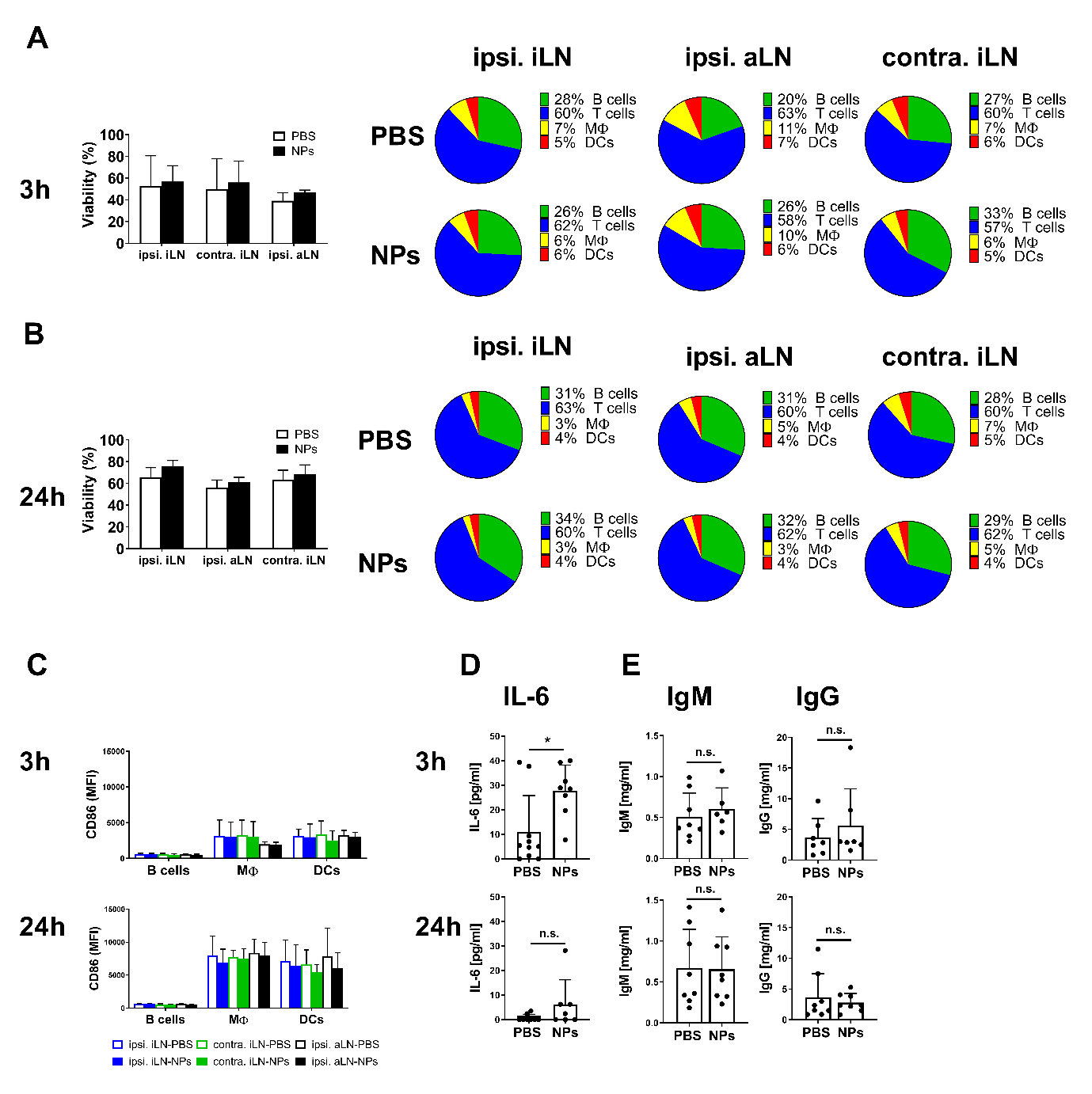


**Figure S3:** Cell viability and immune activation after subcutaneous administration of GNPs. **A)** Total cell viability in different LNs and percentage of different immune cell populations in primary draining LN (ipsilateral inguinal LN, ipsi. iLN), secondary draining LN (ipsilateral axillary LN, ipsi. aLN) or non-draining LN (contralateral inguinal LN, contra. iLN) **(A)** 3h and **(B)** 24 h after subcutaneous injection of 300 µg GNPs in the mouse flank measured by flow cytometry. **C)** Expression of the surface immune activation marker CD86 in different immune cell populations defined in A and B. B cells (CD19^+^/CD3^-^), T cells (CD19-/CD3+), Mφ (CD11b^+^/CD11c^-^), DC: (CD11b^+/-^/CD11c^+^). Production IL-6 **(D)** and total serum IgM and IgG **(E)** concentrations 3 h and 24 h post-injection with GNPs, measured by ELISA. Each dot represents one mouse (n=5-8). Each graph show pooled data of two independent experiments (n=5-8). Error bars in all bar plots: mean ± SD of the pooled data. Data were evaluated by two-way ANOVA followed by Sidak’s multiple comparison post-hoc test in A, B and C and by unpaired Student´s t-test in D and E. n.s: not significant = p>0.05


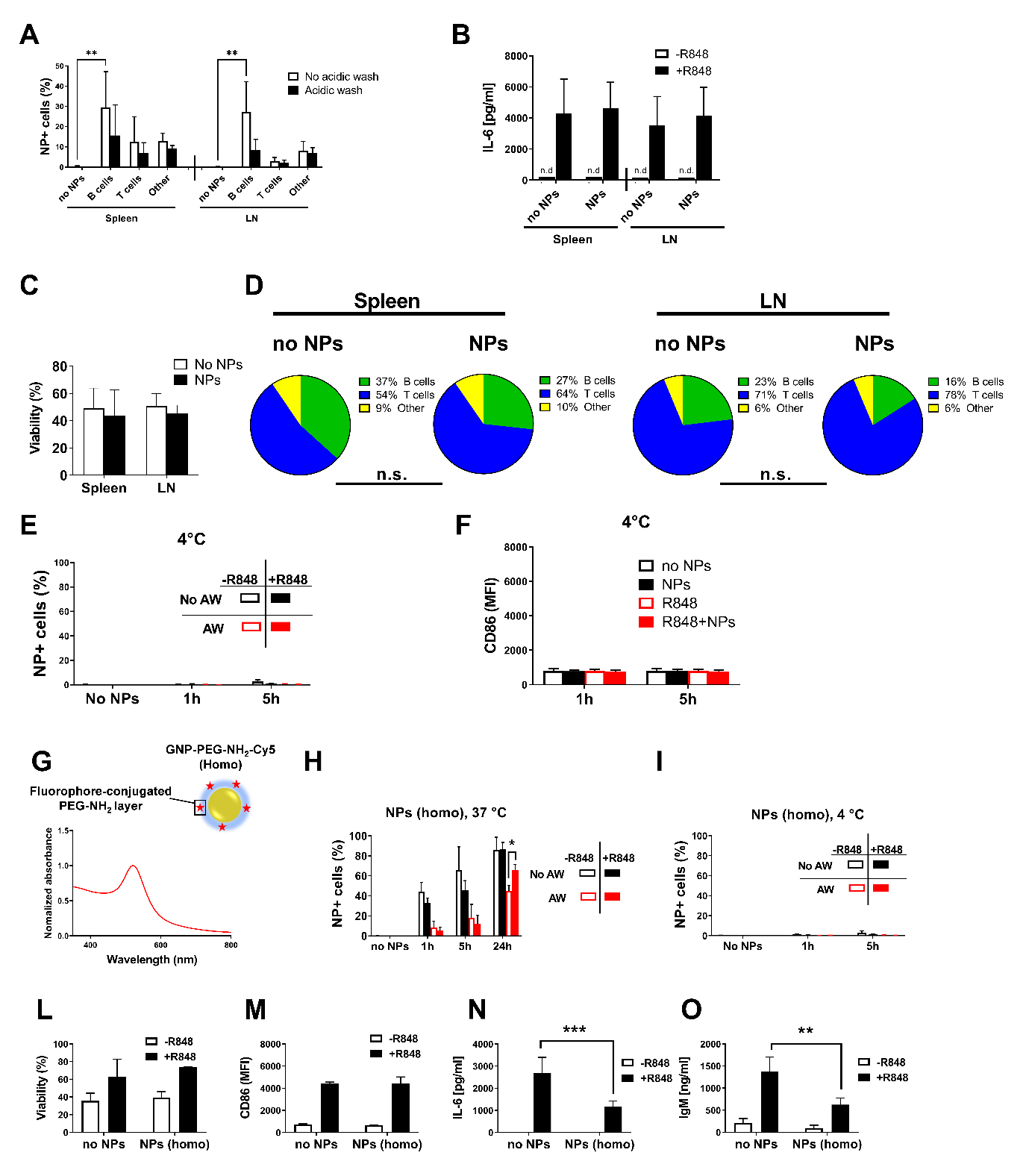


**Figure S4:** Effect of GNPs on splenocytes and Lymph node cells *in vitro*. **A)** Total GNP-B cell association (no acidic wash) or GNP uptake (acidic wash) by different immune cells of spleen and lymph nodes after 24 h of incubation with 20 µg/ml GNPs in the presence or absence of R848 (2 µg/ml) adjuvant, incubated at 37°C and measured by flow cytometry as a percentage of GNP-Cy5 positive cells. **B)** IL-6 concentration in the supernatants produced by splenocytes treated as in A, measured by ELISA. n.d.: not detected. **C)** Cell viability in spleen and lymph node cells 24h after exposure to GNPs measured by flow cytometry using amine-reactive viability dye. **D)** Percentage of immune cells in spleen and Lymph nodes. B cells (CD19^+^/CD3^-^), T cells (CD19^-^/CD3^+^), other (CD19^-^/CD3^-^). **E)** Total GNP-B cell association (no acidic wash) or GNP uptake (acidic wash) by isolated splenic B cells after 1 and 5 h after exposure to 20 µg/ml GNPs in the presence or absence of R848 (2 µg/ml), incubated at 4°C and measured by flow cytometry as a percentage of GNP-Cy5 positive cells. **F)** Expression of CD86 on B cells. **G)** Schematic presentation of fluorescently labeled (Cy5) GNPs coated with a single PEG layer (NH_2_-PEG) (homo-GNPs) and their UV-Vis spectra in water. Total association or uptake of homo-GNPs on isolated splenic B cells after 1, 5 and 24 h of exposure to 20 µg/ml homo-GNPs. B cells were incubated at 37°C **(H)** or at 4°C **(I)** in presence or absence of R848 (2 µg/ml), measured by flow cytometry as a percentage of GNP-Cy5 positive cells. **L)** B cell viability and **(M)** expression of the activation marker CD86, after 24h exposure with homo-GNPs measured by flow cytometry. IL-6 production **(N)** and IgM secretion **(O)** in the supernatants of isolated B cells exposed to homo-GNPs for 24h measured by ELISA. All data show three independent experiments combined (n=3). Data in A, B, C, L, M, N, O were evaluated by two-way ANOVA followed by Sidak’s multiple comparison post-hoc. Data in D, E, F, H, I were analyzed by Tukey’s multiple comparison post-hoc test. n.s.: not significant = p>0.05, **p*<0.05, ***p*<0.01, ****p*<0.001. Error bars in all bar plots: mean ± SD of the pooled data. No acidic was: -AW, with acidic wash: AW.


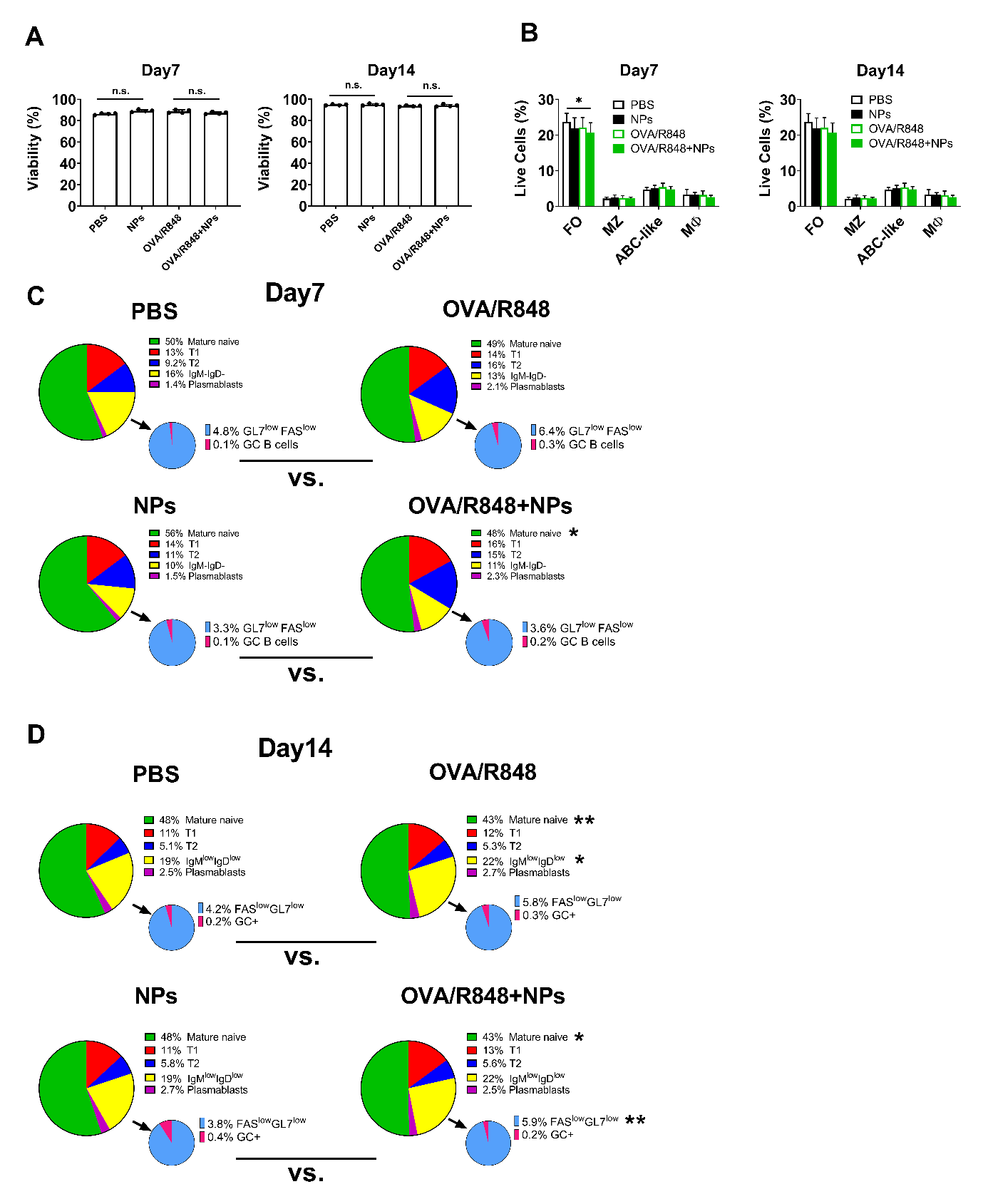


**Figure S5:** Impact of GNPs on splenocytes after OVA immunization. **A)** Viability of splenocytes in C57/BL6 mice 7 days (left panel) and 14 days (right panel) after intravenous injection with 400 µg GNPs and subcutaneous injection with OVA antigen (100 µg)/R848 (10 µg) measured by flow cytometry, using amine-reactive viability dye. Data were evaluated by one-way ANOVA followed by Dunnett´s multi comparison post-hoc test. n.s.: not significant. **B)** Percentage of MZ B cells, FO B cells and macrophages in the spleen, 7 days (left panel) and 14 (right panel) post-injection with GNPs and OVA/R848. Error bars of all bar plots: mean ± SD. Populations were gated as shown in Figure S9; MZ: marginal zone B cells (CD19^+^/CD21^+^/CD23^-^), FO: follicular B cells (CD19^+^/CD21^+^/CD23^+^), ABC-like B cells (CD19^+^/CD21^-^/CD23^-^), Mφ (CD11b^+^/CD11c^-^). Percentage of different splenic B cell subsets upon injection with GNPs and OVA/R848 7 days **(C)** and 14 days **(D)** post-injection with GNPs and OVA/R848. Populations are gated as shown in Figure S12; T1: transitional 1 (CD19^+^/IgM^high^/IgD^low^), T2: transitional 2 (CD19^+^/IgM^high^/IgD^high^), mature naive B cells (CD19^+^/IgM^low^/IgD^high^), IgM^-^IgD^-^ (CD19^+^/IgMl^ow^/IgD^low^), (plasmablasts (CD19^+^/CD138^+^), GL7^low^ FAS^low^ B cells (CD19^+^/IgM^low^/IgD^low^/GL7^low^/FAS^low^), GC B cells: germinal center B cells (CD19^+^/IgM^low^/IgD^low^/GL7^+^/Fas^+^). Representative data of one experiment (day 7; n=4) and two pooled experiments (day 14, n=8). Data in B, C and D were evaluated by two-way ANOVA, followed by Tukey’s multiple comparisons post-hoc. For C and D only the results of PBS vs OVA/R848 and NPs vs OVA/R848+NPs comparisons are shown. *p<0.05, **p<0.01.

**
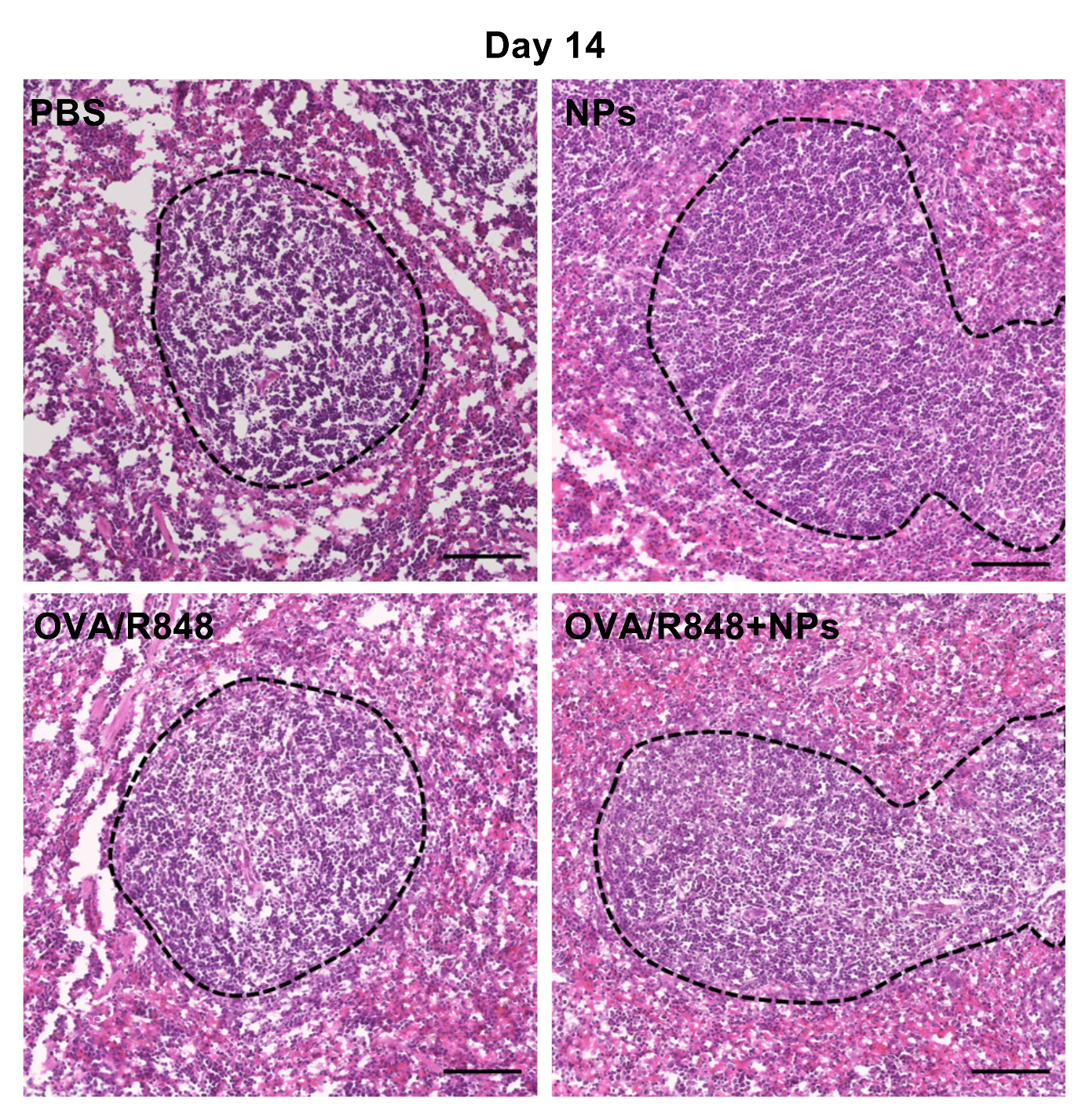
Figure S6:** Spleen tissue 14 days after OVA immunization and GNP injection. Representative images of spleen 14 days after injection with 400 µg GNPs and OVA antigen (100 µg)/R848 adjuvant (10 µg) obtained by slide scanning microscope, using hematoxylin and eosin staining. Scale bar: 100 μm. Dashed lines indicate indicates B cell follicles.


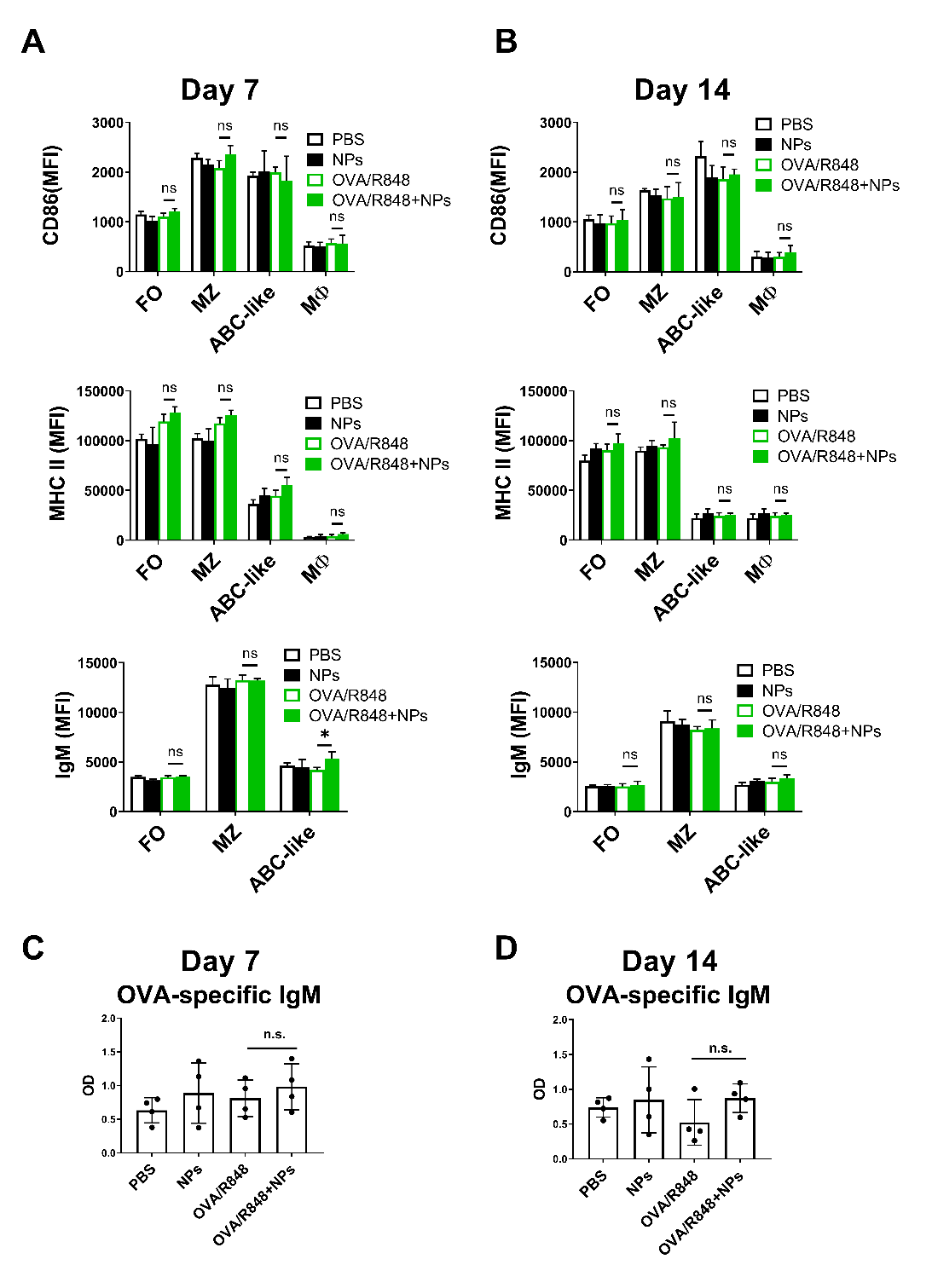


**Figure S7:** Impact of GNPs on B cell activation and antibody production in OVA-immunized mice. Surface activation markers on MZ, FO B cells and macrophages 7 days (**A)** and 14 days (**B)** after injection of C57/BL6 mice with 400 µg GNPs and OVA antigen (100 µg)/R848 adjuvant (10 µg). Data show one of two biological experiments (n=4). Data are evaluated by two-way ANOVA, followed by Tukey’s multiple comparisons post-hoc test. n.s.: not significant=p>0.05, *p<0.05. Populations are gated as shown in Figure S1; MZ: marginal zone B cells (CD19^+^/CD21^+^/CD23^-^), FO: follicular B cells (CD19^+^/CD21^+^/CD23^+^), Mφ (CD11b^+^/CD11c^-^). Production of OVA-specific IgM 7 days **(C)** and 14 days **(D)** after injection with GNP and OVA/R848. Data show one representative experiment of two. Each dot represents cells from one mouse (n=4). Error bars of all bar plots: mean ± SD. Data were evaluated by one-way ANOVA, followed by Dunnett´s multi comparison post-hoc test. n.s.: not significant=p>0.05.

**
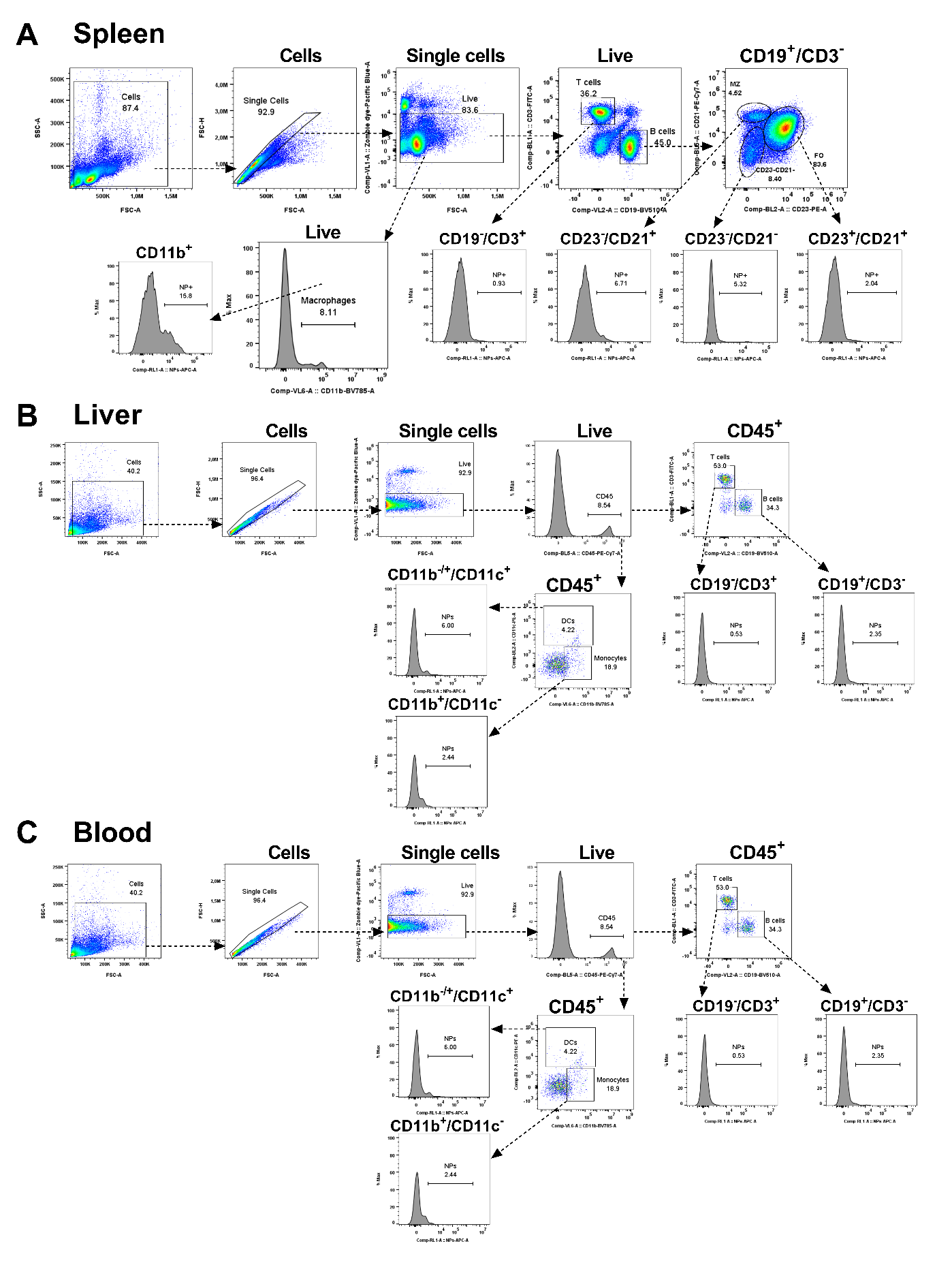
**

**Figure S8:** Gating strategy for different organs in *in vivo* experiments. **A)** Gating strategy for splenocytes after *in vivo* exposure to GNPs. First, leukocytes were gated and doublets were excluded, then live cells were identified by an amine-reactive viability dye. Different cell populations were defined as followed: B cells (CD19^+^/CD3^-^), T cells (CD19^-^/CD3^+^), other (CD19^-^/CD3^-^). B cells were further gated for marginal zone B cells (MZ: CD23^-^/CD21^+^), follicular B cells (FO: CD23^+^/CD21^+^) and ABC-like B cells (CD23^-^/CD21^-^). Finally, defined cell populations were gated on GNP-Cy5 positive cells (NP+). **B)** Gating strategy for liver cells after *in vivo* exposure to GNPs. Cells were gated and doublets excluded, followed by exclusion of dead cells. Next, leukocytes (CD45^+^) were gated, followed by gating of different cell populations as followed: B cells (CD19^+^/CD3^-^), T cells (CD19^-^/CD3^+^), Mφ (CD11b^+^/CD11c^-^), DC (CD11b^+/-^/CD11c^+^). Finally, defined cell populations were gated on GNP-Cy5 positive cells (NP+). **C)** Gating strategy for blood leukocytes after *in vivo* exposure to GNPs. Leukocytes were first gated and single cells were selected. After exclusion of dead cells, leukocytes (CD45^+^) were gated and different cell populations were defined as followed: B cells (CD19^+^/CD3^-^), T cells (CD19^-^/CD3^+^), Mφ (CD11b^+^/CD11c^-^), DC (CD11b^+/-^/CD11c^+^). Finally, defined cell populations were gated on GNP-Cy5 positive cells (NP+).


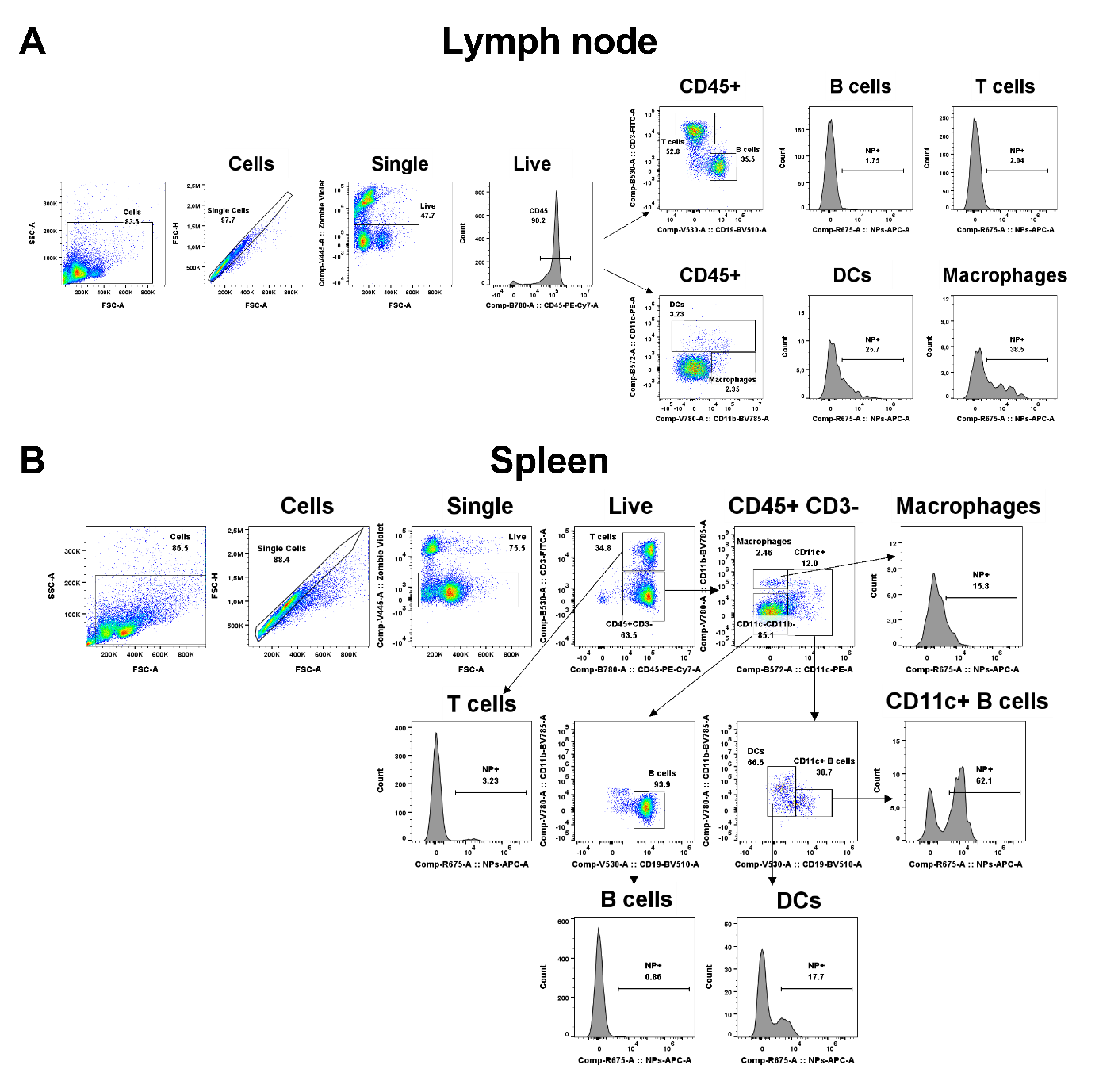


**Figure S9:** Gating strategy for lymph nodes and spleen isolated after subcutaneous injection of GNPs. **A)** Gating strategy for Lymph node cells after *in vivo* exposure to GNPs. Leukocytes were selected, followed by gating on single cells. Next, live cells were determined by an amine-reactive viability dye. Leukocytes were identified as CD45^+^ cells, then different cell populations were defined as followed: B cells (CD19^+^/CD3^-^), T cells (CD19^-^/CD3^+^), Macrophages (Mφ) (CD11b^+^/CD11c^-^), Dendritic cells (DCs) (CD11b^-^/CD11c^+^). Finally, defined cell populations were gated on GNP-Cy5 positive cells (NP+). **B)** Gating strategy for splenocytes after *in vivo* exposure to GNPs. Leukocytes were selected, followed by exclusion of doublets. Next, live cells were determined by an amine-reactive viability dye. Leukocytes were identified as CD45^+^ cells, then different cell populations were defined as followed: B cells (CD11b^-^/CD11c^-^/CD19^+^), T cells (CD45^+^/CD3^+^), Macrophages (Mφ) (CD11b^+^/CD11c^-^), Dendritic cells (DCs) (CD19^-^/CD11c^+^), ABC-like B cells (CD11c+ B cells: CD11c^+^/CD11b^-^/CD19^+^). Finally, defined cell populations were gated on GNP-Cy5 positive cells (NP+).

**
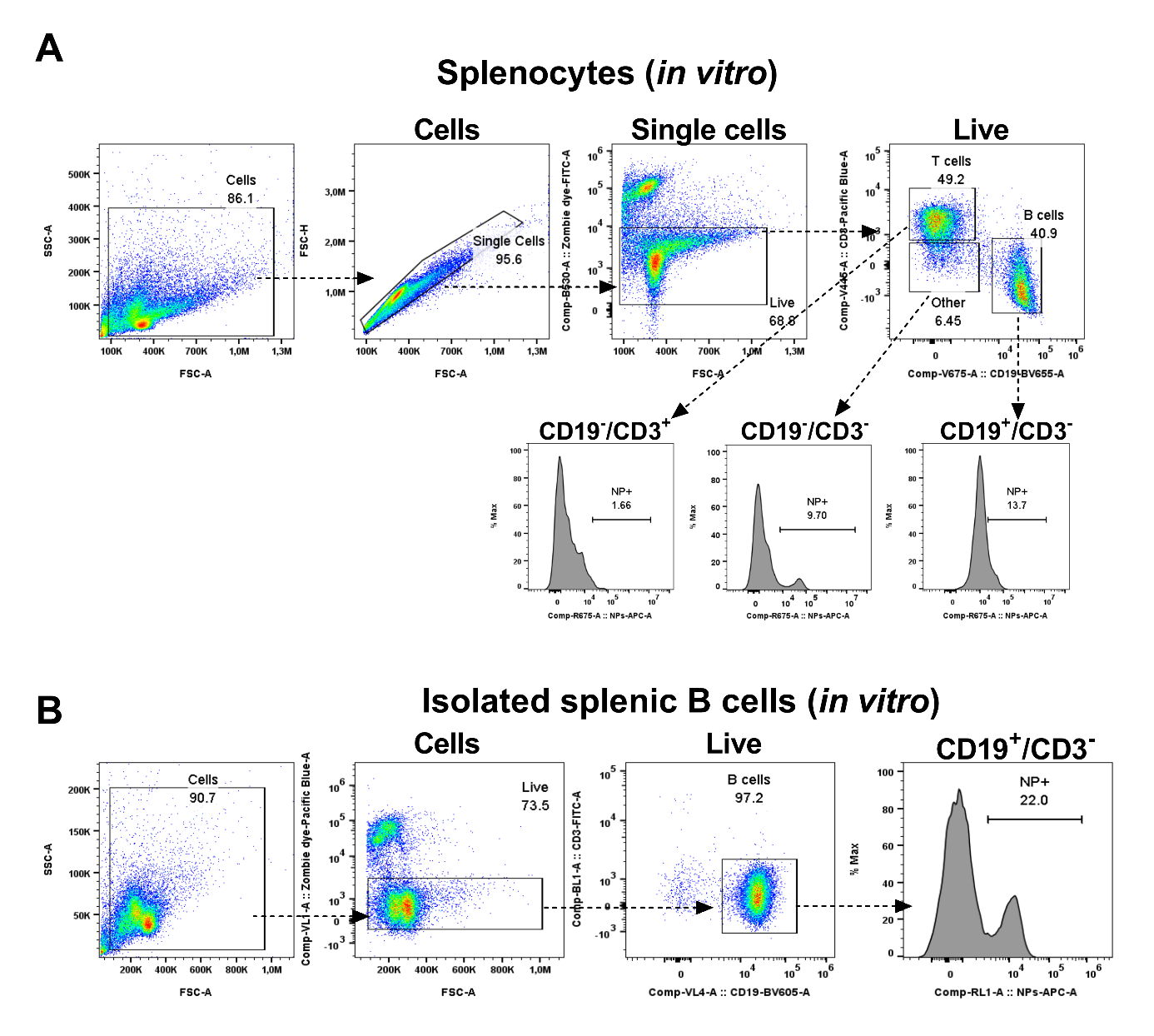
Figure S10:** Gating strategy for *in vitro* experiments. **A)** Gating strategy for splenocytes after *in vitro* exposure to GNPs. All cells were first gated followed by gating on single cells only. Next, live cells were determined by an amine-reactive viability dye. Different cell populations were defined as followed: B cells (CD19^+^/CD3^-^), T cells (CD19^-^/CD3^+^), other (CD19^-^/CD3^-^). Finally, GNP-Cy5 positive cells were gated (NP+). **B)** Gating strategy for isolated splenic B cells after *in vitro* exposure with GNPs and B cell purity. All cells were first gated followed by gating on live cells (amine-reactive viability dye). B cells were defined as CD19^+^/CD3^-^ with >96% purity. Finally, GNP-Cy5 positive B cells were defined (NP+).

**
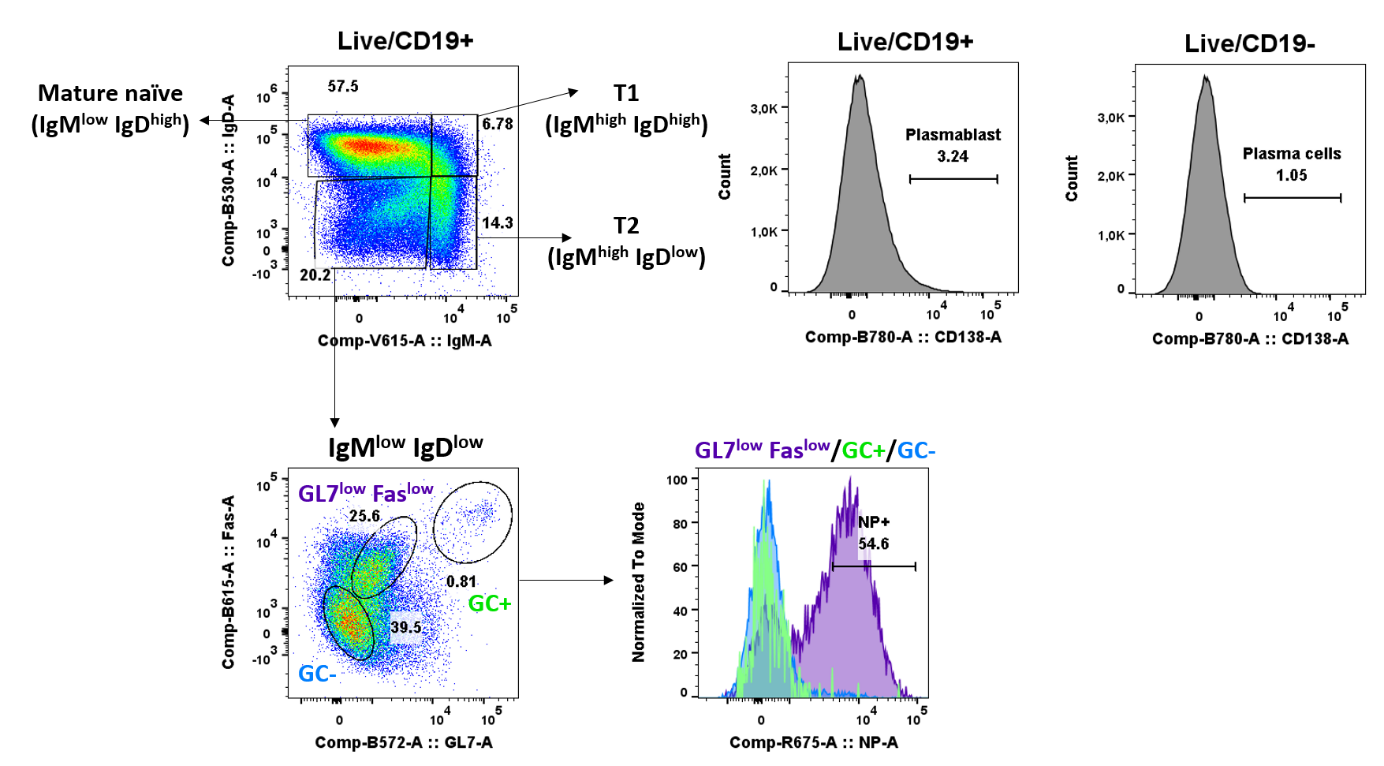
**

**Figure S11:** Gating strategy to determine different populations of B cells in the spleen after *in vivo* exposure to GNPs. After the exclusion of cell debris, dead cells and doublets, CD19+ B cells were defined. CD19^+^ B cells were further gated for T1: transitional 1 (CD19^+^/IgM^hi^/IgD^low^), T2: transitional 2 (CD19^+^/IgM^hi^/IgD^hi^), mature naive B cells (CD19^+^/IgM^low^/IgD^int^), plasmablasts (CD19^+^/CD138^+^), plasma cells (CD19^-^/CD138^+^), germinal center positive (GC^+^) B cells (CD19^+^/IgM^low^/IgD^low^/GL7^+^/Fas^+^), GL7^low^/Fas^low^ cells (CD19^+^/IgM^low^/IgD^low^/GL7^low^/Fas^low^) and germinal center negative (GC^-^) B cells (CD19^+^/IgM^low^/IgD^low^/GL7^-/^Fas^-^). Defined cell populations were further gated on GNP-Cy5 positive cells. A representative example for GC^+^ GNP-Cy5^+^, GL7^low^/Fas^low^ GNP-Cy5^+^ and GC^-^ GNP-Cy5^+^ positive B cells is shown.


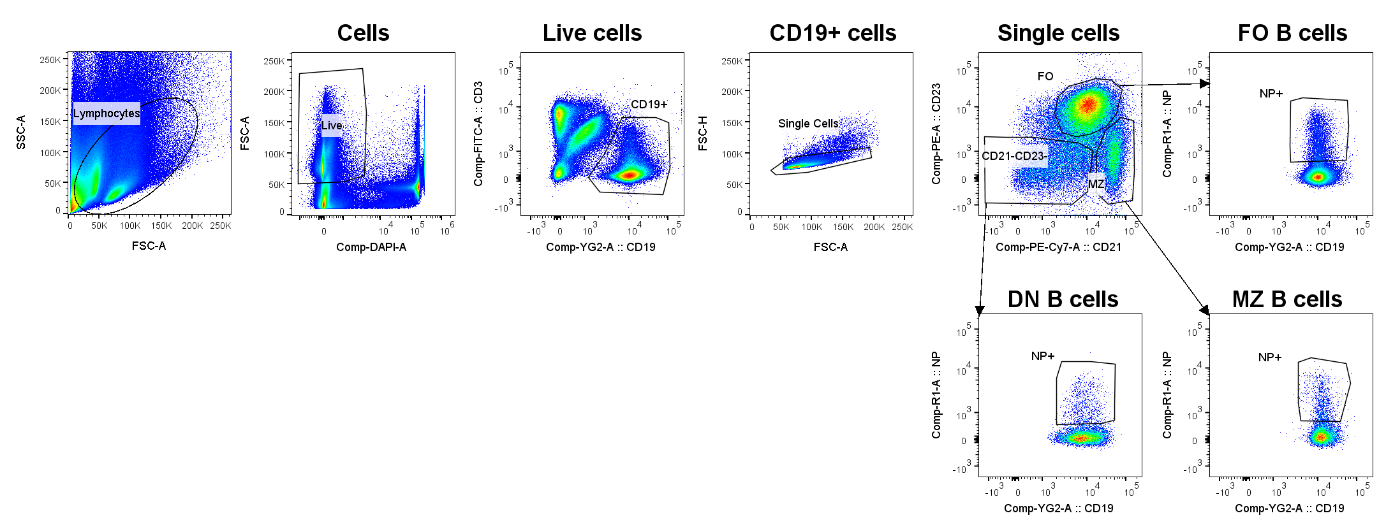


**Figure S12:** Gating strategy for sorting of GNP+ B cells. C57/BL6 mice were injected intravenously with 400 µg GNPs, immediately followed by injection of a mixture of OVA antigen (100 µg) and R848 adjuvant (10 µg). After 14 days, splenocytes were collected and GNP-Cy5+ B cells were isolated by fluorescence-activated cell sorting with the following gating strategy. After the exclusion of cell debris and dead cells, B cells were defined as CD19^+^/CD3^-^ cells. After exclusion of doublets, the different subpopulations of B cells were identified as follicular cells (FO: CD21^+^/CD23^+^), marginal zone cells (MZ: CD21^+^/CD23^-^), CD21^-^ CD23^-^ cells (DN: double negative, CD21^-^/CD23^-^). GNP-Cy5+ (NP+) cells from each subpopulation were finally collected.
